# Cargo Secreted by the Type IX Secretion System of *Porphyromonas gingivalis* Are Tethered to *O‐Lipopolysaccharides* via a Pentasaccharide Linker

**DOI:** 10.1002/mbo3.70296

**Published:** 2026-04-16

**Authors:** Paul D. Veith, Michael G. Leeming, Yu Yen Chen, Eric C. Reynolds

**Affiliations:** ^1^ Oral Health Cooperative Research Centre, Melbourne Dental School, Bio21 Institute The University of Melbourne Parkville Victoria Australia; ^2^ Melbourne Mass Spectrometry and Proteomics Facility, Bio21 Molecular Science and Biotechnology Institute University of Melbourne Melbourne Victoria Australia

**Keywords:** glycoprotein, glycosylation, lipopolysaccharide, mass spectrometry, *Porphyromonas gingivalis*, type IX secretion system

## Abstract

The Gram‐negative oral pathogen, *Porphyromonas gingivalis*, uses the Type IX Secretion System (T9SS) to secrete major virulence factors (cargo proteins) and anchor them to the cell surface via a novel linking sugar, 2‐N‐seryl, 3‐N‐acetylglucuronamide (SAGA), which is a component of a specific type of lipopolysaccharide, A‐LPS. The reported structure of the polysaccharide component (A‐PS) was a repeating phosphorylated mannan whereas the PS of conventional *O‐*LPS (*O‐*PS) is a repeating Gal‐Glu‐Rha‐GalNAc unit. Here, we have performed extensive mass spectrometric analyses of cargo protein‐linked LPS with and without proteinase K treatment to determine the structure of A‐LPS. Limited acid hydrolysis of the PS backbone with trifluoromethanesulfonic acid enabled long PS fragments linked to cargo‐derived peptides to be identified for the first time. Unexpectedly, rather than finding A‐PS units, up to eleven *O‐*PS repeating units were found linked to cargo via a novel pentasaccharide linker designated A‐LS, composed of SAGA‐Hex‐dHex(C_4_H_4_O_3_)(Pent)‐Hex. In addition, samples from a *wzzP/porT* double mutant that produced free truncated *O‐*PS were specifically hydrolyzed to cleave lipid A prior to MS analysis. In these samples A‐LS was found attached to a limited number of *O‐*PS repeating units that in turn were associated with a putative core oligosaccharide that included the LPS‐specific sugar, 3‐deoxy‐d‐manno‐octulosonic acid (Kdo). The proposed structure of A‐LPS explains all 11 genes specific to A‐LPS biosynthesis, and provides the first structural evidence that cargo proteins such as the gingipains are anchored to the cell surface via a complete LPS molecule.

## Graphical abstract

Cargo proteins secreted by the Type IX Secretion System of *Porphyromonas gingivalis* were purified. After proteolysis and partial cleavage of their glycan portions, mass spectrometry analyses led to the identification of novel LPS constituents including a pentasaccharide that linked the cargo to the *O*‐polysaccharide.

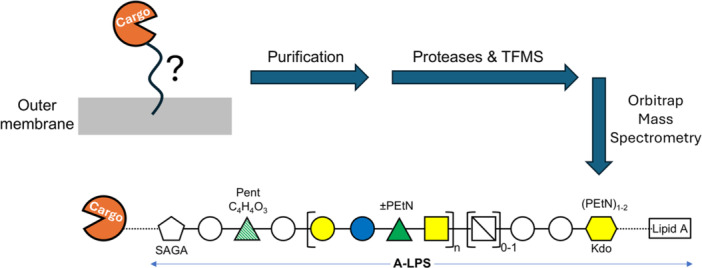

## 1 Introduction

Lipopolysaccharide (LPS), also known as endotoxin, is a prominent cell‐surface antigen in Gram‐negative bacteria. While the exact chemical structure of LPS is variable, these molecules are composed of three distinct domains: (i) Lipid A which consists of a phosphorylated disaccharide esterified with multiple fatty acids and functions to anchor the LPS structure into bacterial outer membranes, (ii) the core oligosaccharide (core), and (iii) the *O*‐polysaccharide (*O*‐PS) also known as the *O*‐antigen which comprises the outermost domain of LPS (Raetz and Whitfield 2002). The core and *O*‐PS components are extremely diverse besides the presence of 3‐deoxy‐d‐manno‐octulosonic acid (Kdo) in the inner core. The sugar elements of the core exhibit structural diversity through modifications including phosphorylation, amino acid and ethanolamine substitution (Klein et al. 2013) and the outer *O*‐PS domain may contain various numbers of repeating units of different sugars (Rocchetta et al. 1999; Liu et al. 2020). The structure and biosynthesis of Kdo_2_‐lipid A is well conserved across Gram‐negative bacteria and is the defining structural unit of LPS (Whitfield and Trent 2014).

The precise molecular composition of the Lipid A domain is important for the endotoxic properties of LPS (Lu et al. 2009), while the composition of the *O*‐PS domain is important for serum resistance (Rocchetta et al. 1999; Liu et al. 2020). In the case of *Porphyromonas gingivalis*, a Gram‐negative anaerobe strongly associated with periodontitis, a chronic and destructive inflammatory disease of the supporting tooth tissues, two types of LPS have been described sharing the same lipid A‐core but different polysaccharides. The first of these is an *O*‐PS shown to have a repeating unit of GalNAc‐Rha‐Glc‐Gal (Paramonov et al. 2001), and the second is anionic polysaccharide (A‐PS) suggested to be a phosphomannan (Paramonov et al. 2005). *O*‐PS was shown to attach to the core at the terminal mannose while A‐PS was attached to the core at a different mannose (Paramonov et al. 2009; Paramonov et al. 2015). The core was reported to be comprised of at least seven mannose residues substituted with phosphoethanolamine (PEtN), a glycerol, an allose and at least one Kdo (Paramonov et al. 2009). *P. gingivalis* lipid A is mostly mono‐phosphorylated and tetra‐ or penta‐acylated, and these different forms differentially modulate the innate immune response (Chen et al. 2011; Herath et al. 2013; Olsen and Singhrao 2018). Furthermore, the acylation level was found to be modulated by hemin concentration (Al‐Qutub et al. 2006).

Antigenically, there are also at least two kinds of *P. gingivalis* LPS. One form is recognized by the monoclonal antibody MAb‐1B5 while silver staining of the same LPS preparations shows a different pattern suggesting the presence of additional forms (Shoji et al. 2013; Shoji et al. 2014; Shoji et al. 2018). Furthermore, the MAb‐1B5 reactive LPS appears to be the “anchor” that enables cargo proteins secreted by the Type IX Secretion System (T9SS) to be retained on the cell surface. MAb‐1B5 was generated using a modified T9SS cargo protein as the immunogen (Curtis et al. 1999). Since these cell‐surface attached cargo proteins are essential for the black pigmentation of colonies grown on blood agar, mutants that lose reactivity toward MAb‐1B5 also lose their colonial pigmentation (Nakayama 2015; Lasica et al. 2017; Veith et al. 2017). The cargo proteins themselves have been cataloged (Veith et al. 2021a) and analyzed extensively due to their importance for a variety of virulence traits (Veith et al. 2022). Most were shown to be modified with a high MW heterogenous molecule consistent with LPS (Veith et al. 2013), which was deduced to be transferred to the C‐terminus of matured cargo by the T9SS sortase, PorU (Glew et al. 2012; Gorasia et al. 2015; Veith et al. 2020). The most studied cargo are a group of cysteine proteinases known as the gingipains which have been shown to be essential for virulence in animal models and able to dysregulate the host immune system (O'Brien‐Simpson et al. 2003; Guo et al. 2010).

LPS preparations that yielded the suggested phosphomannan structure were also positive to MAb‐1B5 in Western blots (Paramonov et al. 2005). When a sample was treated to remove phosphorylation, it also lost its immunoreactivity towards MAb‐1B5 and this was interpreted by the authors to suggest that the phosphomannan‐containing “A‐LPS” contained the MAb‐1B5 epitope while “*O*‐LPS” did not. However, this conclusion is not supported by the cumulative genetic evidence. Extensive searches for genes, especially those encoding glycosyltransferases that cause loss of colonial pigmentation, have been conducted and none have been found to explain the biosynthesis of the phosphomannan. In contrast, recent evidence suggest that all genes found to be specifically required for the production of MAb‐1B5‐reactive LPS (A‐LPS) can instead be assigned to the biosynthesis of an alternative glycan that includes, 2‐N‐seryl, 3‐N‐acetylglucuronamide (SAGA), that links LPS to T9SS cargo proteins (Veith et al. 2020). Six of these gene products, WbpA (PGN_0613, UgdA), WbpB (PGN_0168), WbpE (PGN_1236, PorR), WbpD (PGN0002), VimE (PGN_1055) and VimA (PGN_1056) were shown to be part of the Wbp/Vim pathway that synthesises SAGA (Shoji et al. 2014; Veith et al. 2020). Interestingly, the 7th enzyme in this pathway, WbpS (PGN_1234) amidates the glucuronic acid portion into glucuronamide which is an essential step for reactivity with MAb‐1B5 but not for cargo protein conjugation (Veith et al. 2020). Together, these new data suggests that SAGA rather than phosphomannan is the critical LPS component containing the MAb‐1B5 epitope that anchors T9SS cargo proteins to the cell surface.

The remaining gene products required specifically for the production of A‐LPS are the glycosyltransferases WbaP, GtfC, GtfF and VimF (Shoji et al. 2018). In theory, these could either be involved in the production of phosphomannan, or involved in the transfer of SAGA and additional sugars. The latter appears more likely since WbaP and GtfC show similar function to WbaQ and GtfD respectively which together appear responsible for transferring the first two sugars of *O*‐PS, neither of which are mannose (Shoji et al. 2018). Furthermore, the *vimF* gene is located adjacent to *vimE* and *vimA* (Vanterpool et al. 2005) and is therefore likely to be involved in a transfer involving SAGA.

Here, we test our hypothesis that SAGA is located on a branch stemming from a polysaccharide similar to *O*‐PS and demonstrate that SAGA is part of a novel pentasaccharide element, potentially located at the distal end of a subpopulation of *O*‐polysaccharide chains. We propose a redefinition of “A‐LPS” to be a variant of *O*‐LPS that includes this novel pentasaccharide designated “A‐linking saccharide” (A‐LS) that confers both reactivity to MAb‐1B5, and ability to anchor T9SS cargo.

## 2 Materials and Methods

### 2.1 Bacterial Strains and Growth Conditions

*P. gingivalis* strains were grown on solid medium (TSBHI agar) containing trypticase soy agar (40 g/L), brain heart infusion (BHI, 5 g/L), 5% (v/v) lysed defibrinated horse blood, cysteine hydrochloride (0.5 g/L) and menadione (5 μg/mL), and subsequently in TSBHI broth (25 g/L Tryptic soy, 30 g/L BHI) with haemin (5 μg/mL), cysteine hydrochloride (0.5 g/L) and menadione (5 μg/mL) under anaerobic conditions as previously described (Gorasia et al. 2015). The double mutant lacking functional WzzP (PGN_2005) and PorT was a kind gift from Dr. Mikio Shoji (Shoji et al. 2013).

### 2.2 Purification of T9SS Cargo Proteins

*P. gingivalis* W50 cargo proteins were purified based on a method previously published for the purification of RgpB (“RI‐B”, [Rangarajan et al. 1997]). Cells from a 6‐day culture were removed by centrifugation at 10,000 g for 30 min, 4°C and the cold supernatant was then precipitated by the addition of ammonium sulphate to 85% saturation. Precipitated proteins were collected by centrifugation at 10,000 g for 60 min, 4°C and washed in acetate buffer (50 mM sodium acetate, pH 5.3) with 0.0055% myristyl sulfobetaine (SB3‐14). The pellet was solubilized in 1% SB3‐14 in acetate buffer, centrifuged to remove particulates, and separated by anion‐exchange chromatography using a Q‐Sepharose column equilibrated in 20 mM Bis‐Tris, 5 mM CaCl_2_, 50 mM NaCl, and 0.05% SB3‐14, pH 6, and eluted with a linear gradient of 50 to 500 mM NaCl. Fractions (2 mL) were collected.

Selected fractions were precipitated with 13% trichloroacetic acid (TCA), washed with ice‐cold acetone and subjected to SDS‐PAGE using a 15‐well 10% polyacrylamide Bis‐Tris gel with morpholinepropanesulfonic acid (MOPS) buffer (Thermo Fisher Scientific) under reducing conditions and stained with Coomassie blue. Q‐Sepharose fractions 34–35 were pooled as “F1” and fractions 39‐40 as “F2.” F1 and F2 were concentrated to ~300 μL using a 10 kDa molecular weight cutoff (MWCO) membrane, topped up to 1 mL with deionized water and TCA precipitated as above. The precipitates were resuspended in 50% acetonitrile (CH_3_CN) in 0.1% aqueous trifluoroacetic acid (TFA) and freeze‐dried until completely dry prior to deglycosylation.

### 2.3 Proteinase K Digestion of F1 and F2

Additional F1 and F2 samples from a second Q‐Sepharose separation, were similarly concentrated and precipitated. These samples were treated with 50 μL of denaturing solution (1% SDS, 50 mM Tris pH 7.5, 10 mM DTT) at 100°C for 10 min and then diluted fourfold into proteolysis buffer (50 mM Tris, pH 7.5, 5 mM CaCl_2_) to which Proteinase K (50 µg/mL) was added. Proteolysis was conducted at 50°C overnight. The digested sample was cleared by centrifugation at 14,000 g for 5 min and filtered through a 3 kDa MWCO membrane (Amicon Ultra‐4) at 7000 x g, 15°C to remove buffer components, small peptides and amino acids. The filter was initially topped up to the maximum volume of 4 mL, concentrated to < 100 µL, and washed with two full volumes of water. Each concentration cycle took approximately 40 min. The final concentrate was freeze‐dried until completely dry and deglycosylated as described below.

### 2.4 Deglycosylation With Trifluoromethanesulfonic Acid (TFMS)

Samples were deglycosylated with TFMS and neutralized as previously described (Veith et al. 2020). The deglycosylated cargo proteins were recovered by TCA precipitation and separated by SDS‐PAGE as described above. Selected protein bands were digested in‐gel with trypsin as previously described (Gorasia et al. 2015).

For the proteinase K treated samples, recovery of the patially deglycosylated glycans after deglycosylation was achieved as follows. The whole of the pyridine‐treated reaction mix (200 µL) was diluted to 2 mL with deionised water. The precipitate formed was removed by centrifugation at 14,000 x g for 10 min, and the supernatant was cleaned by solid‐phase extraction using Sep‐Pak C18 Plus Light (Waters) according to the manufacturer's protocol with elution in 50% CH_3_CN. The eluted material was dried in a vacuum centrifuge and prepared for MS analysis. For samples designated 0 min deglycosylation, the reaction incubation step at −20°C was omitted. The limited reaction would have occurred while the TFMS solution was added to the sample. While this step is performed on a bed of dry ice/ethanol, some degree of deglycosylation was nevertheless evident.

### 2.5 Mild Acid Hydrolysis

Washed cell envelope fractions were obtained from *wzzP/porT* according to our published protocol (Gorasia et al. 2015) and resuspended in 30 mM sodium acetate, pH 4.5 with the aid of a sonication probe (model CPX 750, Cole Parmer) fitted with a 3 mm microtip and employed at minimum power amplitude (19%) to obtain a suspension of fine particles. The suspension was heated at 100°C for 1 h to cleave Lipid A. The residual membranes and insoluble Lipid A were then removed by ultracentrifugation at 150,000 x g for 2 h. The supernatant containing the released polysaccharides was filtered through a 10 kDa MWCO membrane (Amicon Ultra‐4) at 7000 x g, 15° C to remove proteinaceous materials, while the filtrate containing the target polysaccharides was cleaned by solid‐phase extraction, dried and prepared for MS analysis as described above.

### 2.6 Mass Spectrometry

Samples were resuspended in 2% CH_3_CN, 0.1% TFA and separated using a two‐column chromatography setup composed of a PepMap100 C18 20 mm × 75 μm trap and a PepMap C18 500 mm × 75 μm analytical column (Thermo Fisher Scientific). Samples were concentrated onto the trap column at 5 μL/min for 6 min with buffer A (0.1% formic acid) and then infused into an Orbitrap mass spectrometer (see types below) at 300 nL/min via the analytical column using a Dionex Ultimate 3000 UHPLC (Thermo Fisher Scientific). For the trypsin/TFMS treated samples, a 60 min gradient was employed with the concentration of buffer B (80% CH_3_CN, 20% water) varying from 3% to 50%. For Proteinase K/TFMS treated samples, buffer B increased from 3% to 23% in 29 min and then from 23% to 40% in 10 min. For the samples hydrolyzed with mild acid (lipid A removal), buffer B increased from 3% to 30% in 60 min.

For all experiments, the mass spectrometer was operated in a data dependent mode, automatically switching between the acquisition of an Orbitrap MS scan (60,000 resolution) every 3 s (or 2 s for FAIMS experiments) and Orbitrap MS/MS HCD scans of precursors (NCE 30%, maximal injection time of 60 ms, with an AGC of 200% and a resolution of 30,000). Dynamic exclusion (exclude after 2 times within 90 s window) was employed for both trypsin and proteinase K digested samples. When employed, CID scan settings were NCE 30%, maximal injection time of 50 ms, AGC of 200%, a resolution of 15,000 or 30,000 and 3–5 microscans.

For the trypsin/TFMS treated samples, stepped FAIMS experiments were conducted in an Orbitrap Fusion Lumos Tribrid (Thermo Fisher Scientific), switching between CV values of −25 and −45. The MS scan was from *m/z* 500–2000 and ions of charge 1–8 were included. Precursor ions were prioritized according to highest *m/z*. CID scans were triggered by the detection of glycan fragment ions of *m/z* 181.0605, 199.071, 303.1295 or 491.2466 if at least one was detected in the top 20 most intense product ions.

For the Proteinase K/TFMS treated samples (F1 and F2), an Orbitrap Eclipse (Thermo Fisher Scientific) without FAIMS was used. The MS scan was from *m/z* 375–2000 and ions of charge 1–7 were included. Precursor ions were prioritised according to highest *m/z*. CID scans were triggered by the detection of glycan fragment ions of *m/z* 181.0605, 303.1295, 208.036, 204.0867 if at least one was detected in the top 20 most intense product ions. These settings were employed for the global experiments used for MaxQuant quantitation. Further experiments with the same samples were conducted to fragment a larger number of precursors. Each sample (F1 and F2) was run three times, with the precursor selection range varying from (1) 700–1000, (2) 1000–1300 and (3) 1300–1700. Also, precursor ions were prioritised according to highest intensity.

For mild acid‐hydrolyzed samples, the Orbitrap Fusion Lumos Tribrid instrument was used without FAIMS. The MS scan was from *m/z* 500–2000 and ions of charge 1–8 were included. Dynamic exclusion was not applied. The precursor selection range was *m/z* 700–2000 with ions prioritised according to highest intensity. CID scans were triggered by the detection of glycan fragment ions of *m/z* 181.0605, 303.1295, 208.036, 204.0867, regardless of their intensity. In an additional experiment, sensitivity to larger glycans was achieved by excluding singly charged precursors. For MS^3^ analysis, initial HCD (MS^2^) scans were followed by MS^3^ scans (both CID and HCD) upon the detection of Kdo‐related ions at *m/z* 485.093 or 424.040.

Kdo samples (2‐Keto‐3‐deoxyoctonate ammonium salt, Merck) were analyzed using a Thermo Fisher Scientific Orbitrap ID‐X coupled to a Thermo Fisher Scientific Vanquish UHPLC system. Buffer A was 0.1% formic acid in water and buffer B was 0.1% formic acid in acetonitrile. A solution of Kdo in water (1 μL) was injected into a solvent stream of 50% B at a flow rate of 0.35 mL min^−1^. No chromatographic separation was conducted, and the solvent stream was directed into the H‐ESI source of the mass spectrometer. Positive ions were generated using a spray voltage of 3.5 kV and sheath, auxiliary and sweep gas flow rates of 25, 5, and 0 units respectively. The ion transfer tube was held at 300°C and the vaporiser temperature was 150°C. Mass spectra were collected in the orbitrap analyzer with a resolution of 120k at MS1 level and 30k at MS2 level. For analysis of Kdo fragmentation pathways, targeted MS^2^ spectra were acquired using quadrupole isolation with a width of 1 m/z. CID and HCD spectra were recorded with normalized collision energies of between 20% and 30%. Acquisition proceeded for 1 min following sample injection.

### 2.7 MS Quantification

MS1‐level quantification of the MS data was performed using MaxQuant software. Default settings were used except that Label‐free quantification was disabled. The protein data was ignored and instead the “all peptides” table was used as the raw data. This table provided deisotoped mass and intensity data for each observed compound together with its retention time. The data was analyzed in Excel. Before further analysis, compounds with an intensity of less than 2 × 10^6^ were removed. The target polysaccharides produced higher intensity at lower mass where there was also a lot of untargeted compounds. To filter these out, a mass‐weighted intensity filter (MWIF) was developed. First, the “expected intensity (EI)” modeled on prominent polysaccharide series was calculated as EI = 6 x 10^10^e^−0.009m^, and then the mass‐weighted intensity was determined as the observed intensity divided by the expected intensity (I/EI).

### 2.8 Molecular Formula From Accurate Mass

Molecular formula were deduced from accurate mass data using the mf‐finder tool (https://www.chemcalc.org/mf-finder) (Patiny and Borel 2013).

### 2.9 Glycan Nomenclature

The nomenclature used for drawing and abbreviating sugars was taken from the Symbol Nomenclature for Glycans (SNFG) (https://www.ncbi.nlm.nih.gov/glycans/snfg.html).

## 3 Results

### 3.1 T9SS Cargo Proteins Are Linked to *O*‐PS

*P. gingivalis* W50 cargo proteins were enriched by precipitation of culture fluid and fractionated by anion exchange (Supporting Information S1: Figure S1). The fractions known to correspond to the region where abundant cargo proteins elute were run on SDS‐PAGE (Supporting Information S1: Figure S2). An intense diffuse band at ~70‐80 kDa was particularly prominent in fractions 35‐39. Based on a previous study (Gorasia et al. 2015), this 70‐80 kDa band is known to contain the LPS‐modified forms of the RgpB cargo protein (earlier fractions) and the TapA cargo protein (later fractions). Fractions 34‐35 were pooled as “F1”, while fractions 39‐40 were pooled as “F2”.

In order to detect high molecular weight glycans, partial deglycosylation of F1 and F2 was achieved through only brief (0–20 min) treatment with TFMS and the resulting reaction mixtures were analyzed by SDS‐PAGE (Supporting Information S1: Figure S3). The earliest timepoints that exhibited substantial deglycosylation were 0 min for F1 and 5 min for F2 as indicated by the loss of 70 ‐–80 kDa diffuse bands, and the appearance of prominent bands at ~50 kDa consistent with deglycosylated RgpB and TapA (Figure S3). The bands indicated in Supporting Information S1: Figure S3 were excised and digested with trypsin for analysis by MS. The two cargo proteins that yielded the most relevant data were RgpB in F1 and PG1795 (P27) in F2, due to their high abundance and short C‐terminal peptides of VEGT and KGE respectively. In contrast, the C‐terminal peptide of TapA is long, and as a result provided relatively poor data concerning the glycan modifications.

**Figure 1:**
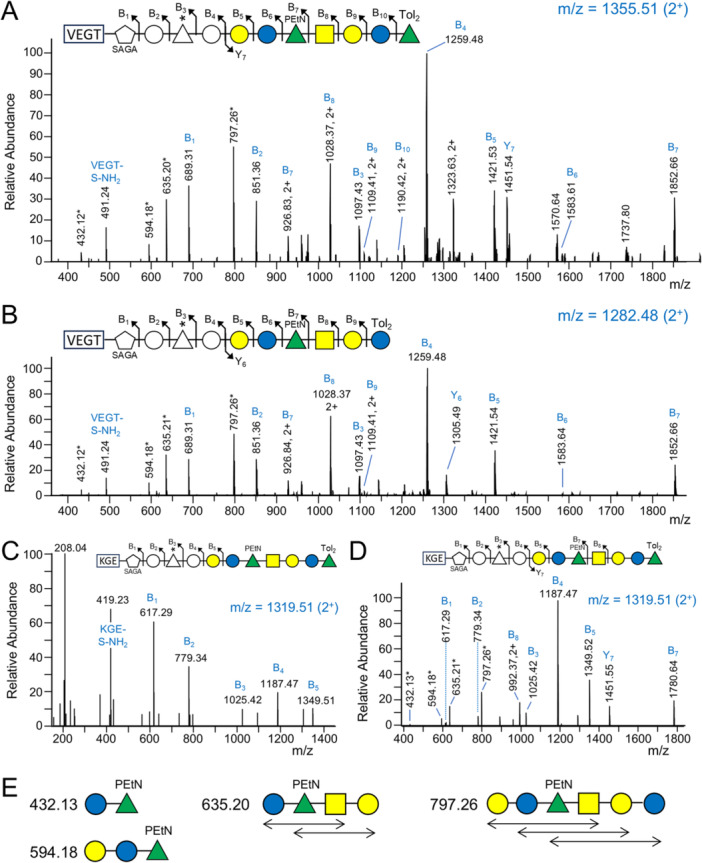
The T9SS cargo protein modification extends to *O*‐PS**.** MS^2^ spectra of partially deglycosylated and trypsin digested T9SS cargo proteins (see Supporting Information S1: Figure S3). For each spectrum, the fragmentation scheme of the glycan is shown above and corresponds to the labeled MS peaks. Peaks labeled with * are internal fragments that are shown in part E. The *m*/*z* value of the precursor ion is shown at the top right of each spectrum in blue text. All fragment ions are single‐charged unless shown otherwise. (A, B). CID spectra of the C‐terminal tryptic peptide of RgpB (VEGT) bonded to the first 11 or 10 sugars, respectively. The spectra are from the analysis of F1, Band 7 (Supporting Information S1: Figure S3). The fragment ion labeled “VEGT‐S‐NH_2_” is the C‐terminal peptide bonded to the serinamide component of SAGA. (C, D). HCD and CID spectra respectively of C‐terminal tryptic peptide of PG1795 (KGE) bonded to the first 11 sugars. The spectra are from the analysis of F2, Band 18 (Supporting Information S1: Figure S3). (E*).* Glycan sequence of common internal fragments (peaks labeled with *). For sugar symbols, see legend in Figure 2. Tol_2_, di‐toluene adduct.

The expected C‐terminal peptide of RgpB is VEGT which exhibits a b^4^ ion at *m/z* at 387.19 followed by expected ions at *m/z* 491.25, 689.31, 851.36 and 1097.43 corresponding to the previously detected glycan additions (Veith et al. 2020). Therefore, to find C‐terminal peptides with longer glycan additions, extracted ion chromatograms (EICs) were plotted using these *m/z* values against all CID MS^2^ spectra. In the first instance, band 7 from F1 was chosen, as it was located slightly higher than the fully deglycosylated RgpB expected in band 8–9 (Supporting Information S1: Figure S3). For example, major peaks were observed at 38.64 min and 41.12 min in EIC traces for *m/z* 851.36 and 1097.43 (Supporting Information S1: Figure S4). The CID spectra at 41.12 min (Figure 1A) and 38.64 min (Figure 1B) showed all four of the expected VEGT peptide sequence ions corresponding to additions of serinamide (S‐NH_2_), N‐acetylglucuronamide (AGA), hexose (Hex) and modified deoxyhexose [dHex(C_4_H_4_O_3_)] consistent with the assignment to glycosylated VEGT.

Peaks at higher mass values suggested the presence of a further Hex residue followed by a series of sugars which we later assigned to the sugars of the *O*‐polysaccharide repeat, namely Gal, Glc, Rha(PEtN) and GalNAc. A partial second *O*‐PS repeat was present in these glycopeptides, terminating with Rha (Figure 1A), or Glc (Figure 1B) with the ditoluene (Tol_2_) adduct at the reducing end as is characteristic of TFMS deglycosylation (Veith et al. 2020). The highest mass peaks were present as doubly charged (2^+^) ions. Similarly, the expected C‐terminal peptide of P27 is KGE which exhibits a b^3^ ion at *m/z* 315.17 followed by expected ions at *m/z* 419.23, 617.29, 779.34 and 1025.42 (Veith et al. 2020), allowing larger modifications to be observed using the same EIC strategy. The same glycan additions were observed as for RgpB, with the longer glycan (terminating with Rha) shown (Figure 1C,D). The HCD spectrum provides improved characterization of low mass ions (Figure 1C), while the CID spectrum more prominently shows higher mass ions (Figure 1D). The doubly charged peak at *m/z* 992.37 indicates the addition of GalNAc. Peaks marked with an asterisk (*) in all spectra correspond to common internal fragments of the glycan portion **(**Figure 1E). Some of these can be explained by multiple structures. For example, the peak with a mass of 797 Da corresponds to Hex_2_‐Rha(PEtN)‐GalNAc and can be explained by three different sequences as marked by the arrows **(**Figure 1E).

While the MS data does not indicate the isomeric form of the sugars, it was evident that the glycan sequence was consistent with the structure of *O*‐PS which has a repeating tetrasaccharide of Gal‐Glc‐Rha(PEtN)‐GalNAc (Paramonov et al. 2001). TFMS is known to favor cleavage either side of dHex residues (Veith et al. 2020; Veith et al. 2021b; Veith et al. 2022a; Veith et al. 2023; Ye et al. 2024) and we therefore analyzed MS data to identify glycan additions that were larger by the mass of the repeating unit (796 Da). Peaks were identified corresponding to 2, 3, 4, and 5 repeating *O*‐PS units linked to RgpB and 2 repeating *O*‐PS units linked to P27. (Table 1).

**Table 1:** MS^1^ matching of C‐terminal peptides of the cargo proteins RgpB and P27 linked to *O*‐polysaccharide.

Glycan sequence	*m/z* (Obs)	*z*	Mass (Obs)	Mass (Calc)	Error (ppm)	CID
VEGT‐Linker‐(*O*‐PS)‐Gal‐Glc‐Tol_2_	1282.483	2	2562.951	2562.9651	−5.3	*
VEGT‐Linker‐(*O*‐PS)‐Gal‐Glc‐Rha‐Tol_2_	1355.513	2	2709.011	2709.0230	−4.2	*
VEGT‐Linker‐(*O*‐PS)_2_‐Gal‐Glc‐Tol_2_	1680.608	2	3359.201	3359.2165	−4.5	*
VEGT‐Linker‐(*O*‐PS)_2_‐Gal‐Glc‐Rha‐Tol_2_	1753.632	2	3505.249	3505.2744	−7.1	*
VEGT‐Linker‐(*O*‐PS)_3_‐Gal‐Glc‐Tol_2_	1386.164	3	4155.470	4155.4679	0.56	—
VEGT‐Linker‐(*O*‐PS)_3_‐Gal‐Glc‐Rha‐Tol_2_	1434.843	3	4301.507	4301.5258	−4.3	—
VEGT‐Linker‐(*O*‐PS)_4_‐Gal‐Glc‐Tol_2_	1651.574	3	4951.700	4951.7193	−3.8	*
VEGT‐Linker‐(*O*‐PS)_4_‐Gal‐Glc‐Rha‐Tol_2_	1700.260	3	5097.758	5097.7772	−3.7	*
VEGT‐Linker‐(*O*‐PS)_5_‐Gal‐Glc‐Tol_2_	1916.984	3	5747.930	5747.9707	−7.0	*
KGE‐Linker‐(*O*‐PS)‐Gal‐Glc‐Tol_2_	1246.477	2	2490.939	2490.9439	−1.8	*
KGE‐Linker‐(*O*‐PS)‐Gal‐Glc‐Rha‐Tol_2_	1319.506	2	2636.997	2637.0018	−1.6	*
KGE‐Linker‐(*O*‐PS)_2_‐Gal‐Glc‐Tol_2_	1644.603	2	3287.191	3287.1953	−1.2	*
KGE‐Linker‐(*O*‐PS)_2_‐Gal‐Glc‐Rha‐Tol_2_	1717.632	2	3433.249	3433.2532	−1.1	—

*CID supportive of cargo linked to *O*‐PS (e.g peaks at 1259, 1852, 797 and 1593) (1187, 1780 for KGE‐).

The MS data derives from the trypsin digested gel bands shown in Supporting Information S1: Figure S3.

### 3.2 The Linked *O‐PS* Contains a Maximum of at Least 11 Repeat Units

To further investigate these glycans, additional portions of F1 and F2 were digested with proteinase K prior to partial deglycosylation with TFMS. Here, the cleaved LPS was the major macromolecule present, and it was possible to meaningfully analyze and quantitate the data using MS^1^ spectra. Label‐free quantification was applied to the deisotoped data which was then presented as plots of mass versus retention time. A mass‐weighted intensity filter (MWIF), favoring ions of higher mass was applied stringently to view the most prominent compounds, and then gradually relaxed to increase the sensitivity of the analysis. There were 1568 compounds above an MWIF threshold of 0.003 (arbitrary units), with almost all falling within a retention time window of 15–65 min and 0–10,000 Da (Figure S5). Prominent diagonal bands were observed that suggest the presence of polymers or related compounds with repeating units (Figure S5). Analysis of the diagonals found clusters of compounds separated by 123 Da consistent with incomplete incorporation of PEtN in *O*‐PS as previously published (Paramonov et al. 2001); furthermore, the clusters were repeated at a spacing of 796 Da in agreement with the mass of the *O*‐PS repeating unit, and indicating that the diagonals reflect varying lengths of *O*‐PS attached to a variable base.

**Figure 2:**
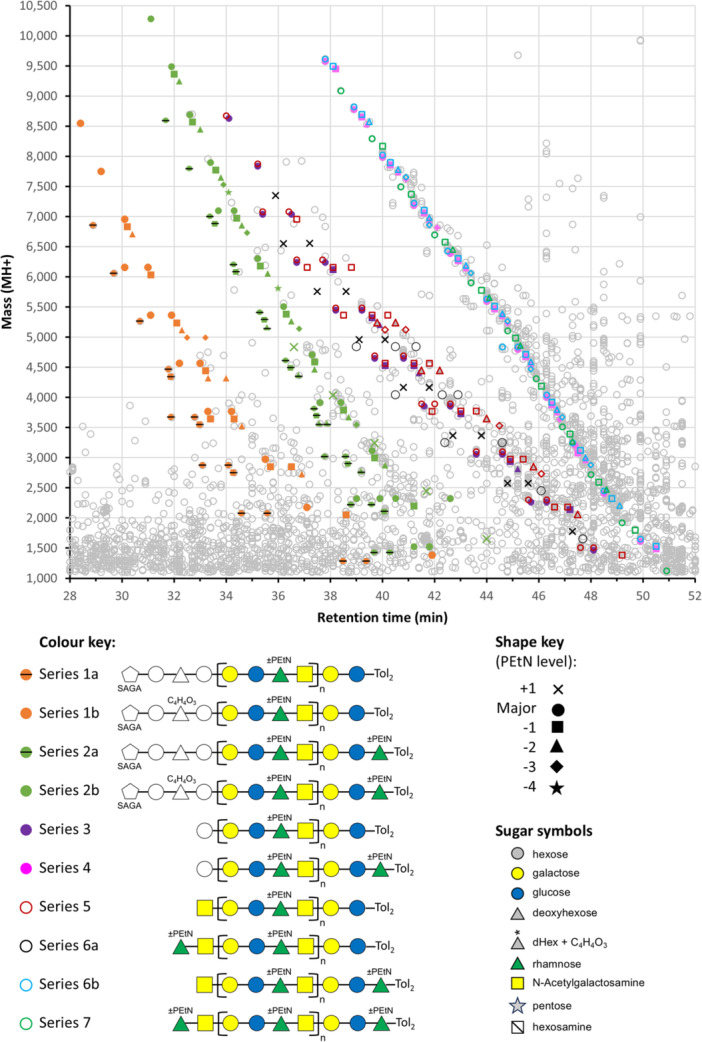
MS^1^ chart showing assignment of compounds to the various polysaccharide series. The chart represents a MaxQuant quantitation of the LC‐MS/MS analysis of F2 showing 3582 compounds above an intensity threshold of 2 × 10^6^ and within the window of 28–52 min and 1100−10,500 Da. The different series are distinguished by color and open or full symbols as shown in the colour key. Within each series, the phosphoethanolamine (PEtN) level is indicated by different shapes, as shown in the shape key. Some compounds appear more than once, but generally only the most intense are shown for clarity. The sugar symbol key is provided as a key for all figures. A summary of the corresponding mass data is shown in Table S1. Tol_2_, di‐toluene adduct.

**Figure 3:**
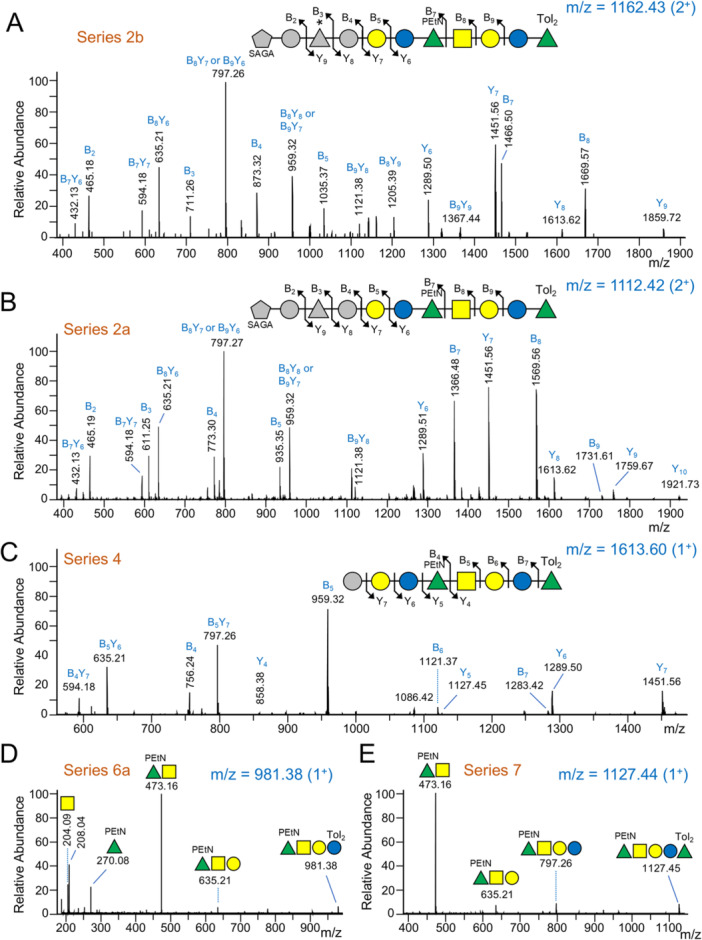
MS^2^ spectra of partially deglycosylated and proteinase K digested T9SS cargo. For each spectrum (A–C), the fragmentation scheme of the glycan is shown above and corresponds to the labeled MS peaks. The scheme is minimalised with as few as possible cleavage points claimed to explain the spectrum. Some internal peaks such as those at *m/z* 797 and 859 can be explained by more than one glycan sequence within the minimalised scheme. For each spectrum (D, E), MS peaks are directly labeled with the assigned glycan. The m/z value of the precursor ion is shown at the top right of each spectrum in blue text. All fragment ions are singly charged. (A, B). CID spectra of series 2b (AO‐PS with C_4_H_4_O_3_) and series 2a (AO‐PS without C_4_H_4_O_3_) both with 11 sugars. C. CID spectrum of series 4 (partial AO‐PS) with eight sugars. D. CID spectrum of series 6a with four sugars. E. CID spectrum of series 7 with five sugars. A summary of all series can be found in Figure 2. For sugar symbols, see legend in Figure 2. Tol_2_, di‐toluene adduct.

The assignments of the “variable bases” and hence the different diagonals (series) were achieved by tracing them to low mass forms where their accurate mass and MS^2^ spectra could be analysed (Figure 2, Table S1). Series 1 and series 2 were identified as the linking saccharide (SAGA‐Hex‐dHex(C_4_H_4_O_3_)‐Hex) connected through to *O*‐PS with or without Rha at the reducing end (Figure 3A, Figure S6) as observed for RgpB and P27 (above). Variants without the C_4_H_4_O_3_ were also observed at a slightly lower retention time (Figure 3B). Series 3 and 4 exhibited a single hexose linked to *O*‐PS with or without Rha at the reducing end (Figure 3C), while series 5–7 corresponded to the *O*‐PS repeat region with or without Rha at either end (Figure 3D,E). Several series reached 9–10 kDa in mass corresponding to a maximum of 11 repeat units (Figure 2, Table S1).

### 3.3 The Linking Saccharide Includes a Pentose

**Figure 4:**
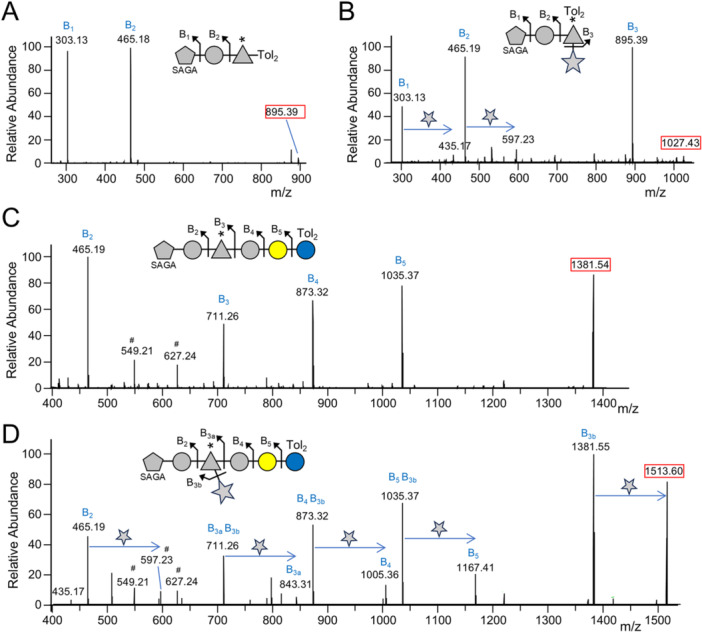
MS^2^ spectra show the presence of pentose in the linker. Further analysis of the proteinase K digested samples. For each spectrum, the fragmentation scheme of the glycan is shown above and corresponds to the labeled MS peaks. The *m/z* value of the precursor ion peak is marked by a red box. All precursor and fragment ions are singly charged. A, B. CID spectra of linker glycans without (A, C) and with (B, D) pentose. See main text and Table 2 for the localization of the pentose residue. It is not clear whether the pentose is bonded to the C_4_H_4_O_3_ portion, or if it is transferred directly to the dHex residue in a C_4_H_4_O_3_‐dependent manner. #Indicates ions that may be produced by dHex scrambling (rearrangement in the gas phase such that dHex is directly bonded to SAGA). For sugar symbols, see legend in Figure 2. Tol_2_, di‐toluene adduct.

As determined in the section above, series 1 and 2 included the linking saccharide which at this point was composed of SAGA‐Hex‐dHex(C_4_H_4_O_3_)‐Hex. A closer analysis of the data revealed the presence of peaks at +132.04 Da in series 1 and 2, suggestive of a pentose. MS^2^ analysis of these peaks (Figure 4B,D) compared to the equivalent peaks lacking pentose (Figure 4A,C) showed a plus pentose peak for each fragment ion, indicating that the putative pentose was labile and present in a branch. While the MS^2^ data indicated that the pentose could be branching from the SAGA residue (see peak at *m/z* 435.17), this could be due to rearrangement in the gas phase. To determine the true location of the branch, we re‐examined the MS^1^‐level data. The pentose was notably specific to series 1 and 2, suggesting that it branches from SAGA or the adjacent Hex and dHex residues. This was confirmed in Figure 4B where the presence of pentose in the SAGA‐Hex‐dHex(C_4_H_4_O_3_) glycan is evident. However, despite an abundant peak at 648 Da (neutral mass) for SAGA‐Hex, a corresponding +132 peak at 780 Da was absent suggesting that the pentose may be linked to the dHex(C_4_H_4_O_3_) residue (Table 2). This was further confirmed by the absence of pentose in Series 1b that lacks C_4_H_4_O_3_ (Table 2). This supports the localization of the pentose to the dHex residue and correlates with the presence of C_4_H_4_O_3_. It is not clear whether the pentose is bonded to the C_4_H_4_O_3_ portion, or if it is transferred directly to the dHex residue in a C_4_H_4_O_3_‐dependent manner.

**Table 2:** Localization of the pentose residue to dHex(C_4_H_4_O_3_).

Glycan sequence	Mass (Calc)	Mass (Obs)	Error (ppm)	MaxQuant Intensity
SAGA‐Hex‐Tol_2_	648.3007	648.3030	3.5	9.5E + 08
(SAGA‐Hex‐Tol_2_) + Pent	780.3429	Not found		—
SAGA‐Hex‐dHex‐Tol_2_	794.3586	794.3621	4.4	5.3E + 08
(SAGA‐Hex‐dHex‐Tol_2_) + Pent	926.4008	Not found		—
SAGA‐Hex‐dHex(C_4_H_4_O_3_)‐Tol_2_	894.3746	894.3784	4.2	9.2E + 08
(SAGA‐Hex‐dHex(C_4_H_4_O_3_)‐Tol_2_) + Pent	1026.4169	1026.4230	5.9	1.2E + 07
SAGA‐Hex‐dHex‐Hex_3_‐Tol_2_	1280.5170	1280.5238	5.3	3.3E + 07
(SAGA‐Hex‐dHex‐Hex_3_‐Tol_2_) + Pent	1412.5593	Not found		—
SAGA‐Hex‐dHex(C_4_H_4_O_3_)‐Hex_3_‐Tol_2_	1380.5331	1380.5328	−0.2	6.6E + 07
(SAGA‐Hex‐dHex(C_4_H_4_O_3_)‐Hex_3_‐Tol_2_) + Pent	1512.5753	1512.5825	4.8	2.6E + 06

*Note:* MS^1^ analysis of proteinase K/TFMS treated sample (MaxQuant output) for the presence or absence of plus pentose peaks.

### 3.4 Putative Linkage of *O*‐PS to a Novel Core Oligosaccharide

Since the *O*‐polysaccharide was reported to be bonded to a core oligosaccharide consisting of a linear mannan substituted with PEtN, glycerol, and Kdo (Paramonov et al. 2009), the data was searched to find evidence of such a connection. While no signals attributable to substituted mannans could be identified, the search for such evidence was complicated by the lack of predictable sites in the core for TFMS cleavage. To circumvent this problem, LPS was cleaved by mild acid hydrolysis to remove lipid A, and rather than using TFMS to cleave the *O*‐polysaccharide, a double mutant lacking both PorT and WzzP was utilised. WzzP is the *O*‐PS chain‐length regulator, and in its absence, *O*‐PS chains are substantially shorter. In the absence of PorT, linkage to T9SS cargo does not occur. Therefore, we expected to find short lengths of *O*‐PS linked to the core oligosaccharide but not linked to protein.

**Figure 5:**
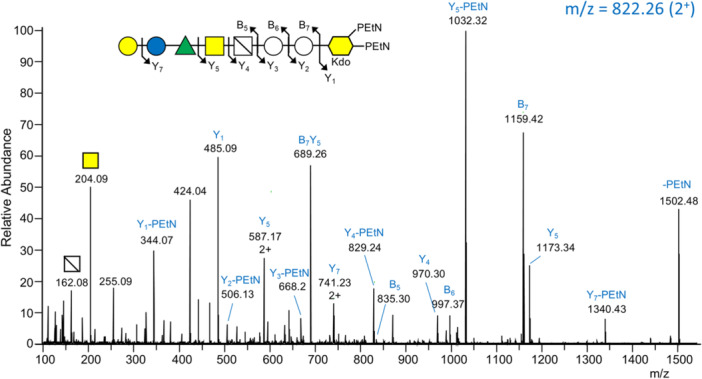
MS^2^ spectrum showing putative linkage of *O*‐PS to core oligosaccharide. A sample obtained from a *porT*/*wzzP* double mutant after lipid A removal. The spectrum is an average of both CID and HCD spectra. The fragmentation scheme of the glycan is shown above and corresponds to the labeled MS peaks. The *m/z* value of the precursor ion is shown at the top right in blue text. All labeled fragment ions are single‐charged unless shown otherwise. For sugar symbols, see legend in Figure 2.

LC‐MS/MS analysis of this sample yielded the identification of glycans with just one or two repeat units with or without the linker and linked to a potentially novel core. The core appeared to mainly consist of Hex_2_ or Hex_2_‐HexN linked to unknown compounds of 343 or 484 Da, which was deduced to be Kdo linked to one or two PEtN units respectively. In the low mass region, MS^2^ spectra typically exhibited strong peaks at *m/z* 344.07 and *m/z* 485.09 corresponding to the Kdo‐PEtN ions, as well as *m/z* 162.08 and *m/z* 204.09 consistent with hexosamine (HexN) and HexNAc respectively. For larger glycans containing the linker, the diagnostic peak at *m/z* 303.13 corresponding to SAGA was observed. The MS^2^ spectrum for the compound with [M + H]^+^ of 1643.507 (*m/z* 822.26, 2^+^) indicated that the Kdo was directly linked to a short oligosaccharide consistent with two Hex and one HexN, which in turn was connected to four sugars with masses consistent with the *O*‐PS repeating unit (Gal‐Glc‐Rha‐GalNAc) (Figure 5). The presence of HexN was found to be variable, being absent in the glycan shown in Figure S7. MS^2^ spectra for three glycans that detail the extension of Kdo through to the linker were obtained (Figure S8). These spectra clearly show the linkage between Kdo and the linker via the *O*‐PS repeating unit and the oligosaccharide consisting of either Hex_2_, or Hex_2_‐HexN. Interestingly, the form of the linker was found to include both the C_4_H_4_O_3_ and pentose residues, with the pentose being highly labile as was also seen in Figure 4. Compounds matching to the mass of glycans that included two *O*‐PS repeating units were found (Table 3), however available fragmentation data in the MS^2^ spectra could not definitively prove their presence.

**Table 3:** Summary of mass data pertaining to the putative core oligosaccharide, with and without MS^2^ verification.

Glycan sequence	Mass (Calc)	Mass (Obs)	Error (ppm)	MaxQuant intensity	MS^2^
[Gal‐Glc‐Rha‐GalNAc]‐Hex‐Hex‐[KDO](PEtN)_2_	1481.434	1481.432	−1.4	1.4E8	Excellent
[Gal‐Glc‐Rha‐GalNAc]‐HexN‐Hex‐Hex‐[KDO](PEtN)_2_	1642.503	1642.500	−2.0	2.2E6	Excellent
[Gal‐Glc‐Rha‐GalNAc]_2_‐Hex‐Hex‐[KDO](PEtN)_2_	2154.677	2154.672	−2.2	4.8E6	Poor
SAGA‐Hex‐dHex*(Pent)‐Hex‐[Gal‐Glc‐Rha‐GalNAc]‐Hex‐Hex‐[KDO](PEtN)	2344.760	2344.751	−3.6	3.6E4	Excellent
SAGA‐Hex‐dHex*(Pent)‐Hex‐[Gal‐Glc‐Rha‐GalNAc]‐HexN‐Hex‐Hex‐[KDO](PEtN)	2505.828	2505.824	−1.9	1.2E5	Excellent
SAGA‐Hex‐dHex*(Pent)‐Hex‐[Gal‐Glc‐Rha‐GalNAc]‐HexN‐Hex‐Hex‐[KDO](PEtN)_2_	2646.848	2646.845	−1.0	3.1E5	Excellent
SAGA‐Hex‐dHex*(Pent)‐Hex‐[Gal‐Glc‐Rha‐GalNAc]_2_‐HexN‐Hex‐Hex‐[KDO](PEtN)	3179.071	3179.051	−6.5		Poor
SAGA‐Hex‐dHex*(Pent)‐Hex‐[Gal‐Glc‐Rha^a^ ^‐^GalNAc]_2_‐Hex‐Hex‐Hex‐[KDO](PEtN)_2_	3444.083	3444.077	−1.6		Poor

^a^In the two copies of *O*‐PS, one of the Rha residues is modified with PEtN.

**Figure 6:**
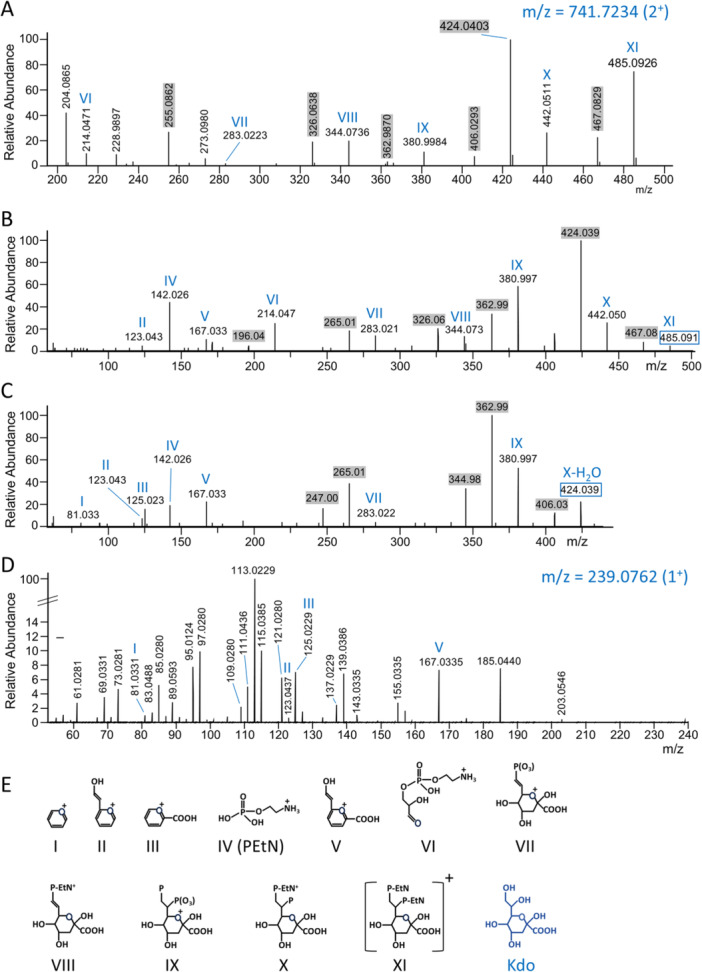
Spectral evidence for Kdo‐(PEtN)_2_. MS^2^ and MS^3^ analysis of (*O*‐PS)_1_‐Hex_2_‐Kdo(PEtN). (A) HCD MS^2^ spectrum of (*O*‐PS)_1_‐Hex_2_‐Kdo(PEtN) at m/z 741.72 (2^+^), showing low mass region from m/z 200‐500. The corresponding full CID spectrum is shown in Supporting Information S1: Figure S7. (B) MS^3^ spectrum of the *m/z* 485 peak (741.72@hcd30; 485.09@hcd28). (C) MS^3^ spectrum of the m/z 424 peak (741.72@hcd30; 424.04@hcd28). (D) HCD MS^2^ spectrum of Kdo (239.076@hcd30). (E) Proposed structures corresponding to peaks labeled with roman numerals. The m/z value of the precursor ion is indicated by a blue box around the labeled mass or else shown at the top right in blue text. All labeled fragment ions are singly charged. Mass labels shaded gray represent potential water loss peaks relative to peaks assigned to a structure.

The Kdo‐PEtN components were at first putatively identified by accurate mass and supposition (Table 4). The low mass region of the MS^2^ spectrum equivalent to that shown in Figure S7 was explored using accurate mass analysis to generate putative molecular formulae from which putative structures were drawn assuming similarity to Kdo (Figure 6A). The mass data accurately matched Kdo substituted with two PEtN units (*m/z* 485), while lower mass peaks accurately matched fragments lacking ethanolamine and phosphoethanolamine units (Figure 6A). To obtain further information, the relation of these fragments to each other was verified by MS^3^ analysis of MS^2^ fragments at *m/z* 485 and 424 (Figure 6B,C). In addition to verification of the PEtN portions, the MS^3^ data provided assurance that additional low mass peaks between *m/z* 81 and 214 were truly derived from the *m/z* 485 and 424 compounds. Several of these putatively matched to the largely dehydrated core Kdo structure missing specific portions including the carboxylic acid and the two carbons (C7 and C8) external to the ring. To gain further confidence in the assignment of Kdo, MS^3^ analyses were also conducted on commercially obtained Kdo. A peak corresponding to protonated Kdo at 239.0762 was observed and fragmented to yield an MS^2^ spectrum (Figure 6D). All four of the fragments proposed to be derived from Kdo (highlighted in Figure 6B,C), were also observed in the commercial Kdo fragmentation spectrum (Figure 6D). The results are therefore consistent with our assignment of the *m/z* 485 compound to Kdo‐(PEtN)_2_.

**Table 4:** Accurate mass analysis of fragments related to Kdo‐PEtN.

Peak label	Peak *m/z* (*z* = 1)	Molecular formula of ion	Error (ppm)	Figure^a^
I	81.0327	[C_5_H_5_O]^+^	−9.8	6C
II	123.0434	[C_7_H_7_O_2_]^+^	−5.3	6C
III	125.0225	[C_6_H_5_O_3_]^+^	−6.6	6C
IV	142.0256	[C_2_H_9_NO_4_P]^+^	−5.4	6C
V	167.0330	[C_8_H_7_O_4_]^+^	−5.3	6C
VI	214.0471	[C_5_H_12_NO_6_P]^+^	−1.9	6A
VII	283.0223	[C_8_H_12_O_9_P]^+^	+3.4	6A
VIII	344.0736	[C_10_H_19_NO_10_P]^+^	−1.5	6A
IX	380.9984	[C_8_H_15_O_13_P_2_]^+^	+0.4	6A
X‐H_2_0	424.0403	[C_10_H_20_NO_13_P_2_]^+^	−0.3	6A
X	442.0511	[C_10_H_22_NO_14_P_2_]^+^	+0.2	6A
XI	485.0926	[C_12_H_27_N_2_O_14_P_2_]^+^	−1.3	6A

*Note:* Putative structures matching the molecular formulae are shown in Figure 6E.

^a^ The figure number corresponds to the spectrum used for the mass data.

## 4 Discussion

**Figure 7:**
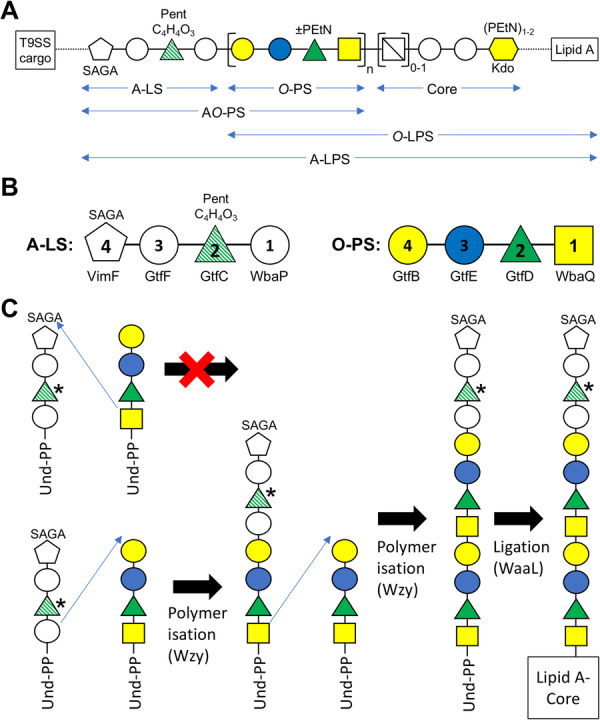
Working model of A‐LPS structure and biosynthesis. (A) Working structural model of A‐LPS showing the A‐linking saccharide (A‐LS) bonded to the *O*‐polysaccharide (*O*‐PS) which in turn is linked to the core oligosaccharide which is expected to be bonded to lipid A. A‐LS is optionally bonded to T9SS cargo proteins via the SAGA linking sugar. (B) Proposed biosynthesis of A‐LS compared to *O*‐PS. The sugars are numbered based on their order of assembly, and the assigned glycosyltransferase for each step is provided underneath. (C) Proposed Wzy‐catalyzed polymerization of A*O*‐PS and its ligation to the lipid A‐core by WaaL. The putative Rha in A‐LS is marked with green stripes. For sugar symbols, see legend in Figure 2. Und‐PP, undecaprenyl pyrophosphate.

In this paper, we extend our previous findings of the structure and biosynthesis of the novel linking sugar, 2‐N‐seryl, 3‐N‐acetylglucuronamide (Veith et al. 2020) which is now abbreviated as SAGA. Previously we found that the T9SS cargo proteins were amide‐linked to SAGA which in turn was found linked to only two additional sugars, a hexose followed by a deoxyhexose. By reducing the extent of TFMS cleavage, the full linker was determined to be a branched pentasaccharide of sequence SAGA‐Hex‐dHex(C_4_H_4_O_3_)(Pent)‐Hex bonded to the *O*‐polysaccharide of sequence Gal‐Glc‐Rha(PEtN)‐GalNAc (Paramonov et al. 2001) with up to 11 repeating units observed. Furthermore, using mild acid hydrolysis conditions known to cleave lipid A from the core, we showed that linker‐*O*‐PS units were bonded to a variable core oligosaccharide comprising HexN‐Hex_2_‐Kdo(PEtN). For reasons explained below, we suggest naming the linker, “A‐linking saccharide” (A‐LS) and when bonded to *O*‐PS, “A‐linked *O*‐PS” (A*O*‐PS), while the whole compound from linker to lipid A is A‐LPS (Figure 7A). “A” originally came from “anionic polysaccharide” (A‐PS), due to it being defined at that time as a phosphomannan that was negatively charged (Paramonov et al. 2005). Since we find that a single copy of A‐LS distinguishes A‐LPS (and not phosphomannan), it is no longer appropriate to maintain the “poly” and “anionic” designations, but it is helpful to maintain the “A” for continuity with existing literature.

The novel finding that A‐LS was covalently bonded to *O*‐PS was conclusive and was demonstrated from both trypsin digested cargo proteins where the C‐terminal peptide remained bonded to the linking sugar (SAGA), as well as proteinase K digested samples where the peptide bond between the protein and SAGA had been cleaved. The presence of *O*‐PS in these structures explains why modified cargo proteins produce a diffuse ladder of bands (Veith et al. 2002; Seers et al. 2006; Shoji et al. 2010; Gabarrini et al. 2018), and also why LPS samples immunoblotted with MAb‐1B5 produce a ladder of bands (Shoji et al. 2013; Shoji et al. 2018). The ladder exhibits the characteristics of typical *O*‐PS that consist of a variable number of repeating units.

In our previous study (Veith et al. 2020), we identified the Wbp/Vim pathway for the biosynthesis of the linking sugar, SAGA, while Shoji et al. (2018) identified the glycosyltransferases required for the step‐wise biosynthesis of the *O*‐PS tetrasaccharide repeating unit, along with the glycosyltransferases WbaP, GtfC, GtfF and VimF required for the production of polysaccharides related to A‐LPS (MAb‐1B5 reactive). Two alternatives were apparent, either A‐LPS uses an A‐type PS *instead* of *O*‐PS, or else it uses just the one type of polysaccharide (*O*‐PS) but with an A‐type branch. The current data conclusively showed the latter, as one copy of the A‐LS was bonded exclusively to *O*‐PS. The proposed difference therefore between *O*‐LPS and A‐LPS is simply the presence of one copy of A‐LS (Figure 7A).

The role of A‐LS is to tether the T9SS cargo proteins to the cell surface to form a surface layer or virulence coat (Chen et al. 2011) which suggests that A‐LS would be attached to *O*‐PS distal to the core. The data presented here appeared to support the A‐LS located at the non‐reducing end of the *O*‐PS chain (distal to the core). Assuming a distal position, the biosynthesis of the A*O*‐PS chain could occur as follows: Firstly, the *O*‐PS and A‐LS units are produced. The und‐PP‐linked *O*‐PS tetrasaccharide (und‐PP‐GalNAc‐Rha‐Glc‐Gal) has been determined to be synthesised by the consecutive actions of WbaQ (initiator), GtfD, GtfE and GtfB (Shoji et al. 2018). Similarly, we propose that the und‐PP‐linked A‐LS (Hex‐dHex(C_4_H_4_O_3_, Pent)‐Hex‐SAGA) is synthesised by the consecutive actions of WbaP (initiator), GtfC, GtfF and VimF (Figure 7B). These four glycosyltransferases are chosen as the only ones identified from a comprehensive analysis of *P. gingivalis* glycosyltransferases to be specific to A‐LPS (Shoji et al. 2018). WbaP, a paralog of WbaQ is first as it is the correct type of glycosyltransferase for the initiation reaction involving transfer of the first sugar to und‐PP (Raetz and Whitfield 2002; Shoji et al. 2013). GtfC is suggested to transfer the 2nd sugar (putative Rha) possibly already bonded to C_4_H_4_O_3_ and Pent, due to its similarity to GtfD and apparent ability to compensate for the lack of GtfD in a *gtfD* mutant (Shoji et al. 2018). GtfF is proposed to transfer the 3rd sugar by process of elimination and finally, VimF is likely to transfer SAGA since the *vimF* gene is part of the *vim* operon responsible for the final steps of SAGA biosynthesis (Vanterpool et al. 2005; Veith et al. 2020).

Secondly, after flipping und‐PP‐linked units across the cytoplasmic membrane, wzy‐dependent polymerization proceeds. The observation of only one copy of A‐LS, and its proposed location at the distal end could be explained by A‐LS only acting as a donor during polymerization and never the acceptor (Figure 7C). In contrast, the *O*‐PS unit must be able to serve as both donor and acceptor in order to polymerize. This phenomenon may be due to Wzy specificity which is poorly understood (Ascari and Morona 2025), or other unknown factors.

Thirdly, after polymerization is terminated, with the length regulated by WzzP (Shoji et al. 2013), the *O*‐PS and A*O*‐PS is ligated to the lipid A core by WaaL. WaaL usually shows little specificity for the *O*‐PS donor beyond the und‐PP portion but is specific to the core acceptor (Raetz and Whitfield 2002). Certainly, in our case, the distal A‐LS is not expected to have an effect as it is the und‐PP end that binds to WaaL, and it is the proximal sugar that is transferred to the core oligosaccharide (Figure 7C) (Ashraf et al. 2022). In a *wzy* mutant, only a single *O*‐PS unit is transferred to the core by WaaL, and in *P. gingivalis*, this product was not recognized by MAb‐1B5 (Shoji et al. 2018). Similarly, our MS data showed that one *O*‐PS unit could be found linked to the core, but the A‐LS was only found connected to the core via at least one *O*‐PS unit (Table 3). This suggests that *P. gingivalis* WaaL may be specific for an *O*‐PS donor and unable to ligate the A‐LS directly to the core.

The proposed structure of A*O*‐PS is consistent with mutant studies. In strains lacking the ability to produce A‐LS, such as the *wbaP*, *gtfC*, *gtfF* and *vimF* mutants, an LPS ladder is detected by silver stain or with the MAb TDC‐5‐2‐1, but not by MAb‐1B5 (Vanterpool et al. 2005; Shoji et al. 2013; Shoji et al. 2018). This indicates that the A‐LS is not essential and that *O*‐LPS can still be synthesised (Figure 7A). In contrast, in the *gtfE* and *gtfB* mutants, LPS ladders are not detected, consistent with these genes catalyzing sugar transfers that are essential for *O*‐PS synthesis (Yamaguchi et al. 2010; Shoji et al. 2018), and demonstrating that without *O*‐PS, the A‐LS is unable to be incorporated into LPS (Figure 7C). This is explained by the A‐LS being present in just one copy and not being able to polymerise to produce a pure “A‐PS”, and as explained above, potentially being unrecognisable to WaaL. Interestingly, the *gtfD* and *wbaQ* mutants appear similar to WT in being able to produce A*O*‐PS, being apparently compensated by *wbaP* and *gtfC* respectively (Shoji et al. 2018). According to our proposed biosynthesis pathway, the *O*‐PS produced in these strains may include the WbaP and GtfC substrates ‐ the first and second A‐LS sugars rather than the usual *O*‐PS sugars. This should be determined in future studies by repeating the MS analysis of LPS isolated from these mutant strains.

Previously, A‐LPS had two defining features. The first being phosphomannan as the anionic polysaccharide component (Paramonov et al. 2005), the second being its reactivity to MAb‐1B5. With the new insights from this study, all the genes known to be specific for the production of MAb‐1B5‐reactive PS are accounted for as being either involved in the biosynthesis of SAGA or the assembly of A‐LS, with none remaining to be assigned to phosphomannan synthesis. Hence it is evident that phosphomannan is not required for MAb‐1B5‐reactivity nor for cargo modification. Therefore, the definition of A‐LPS can no longer include both the presence of phosphomannan and Mab‐1B5 reactivity. Since MAb‐1B5 reactivity has been used in a large number of studies to functionally characterize LPS‐modified cargo and the genes required for A‐LPS synthesis, we have decided to prioritize the property of MAb‐1B5 reactivity and exclude phosphomannan from our new definition of A‐LPS.

Until now, the association of T9SS cargo proteins with A‐LPS has been implied by the laddered bands and the common reactivity to MAb‐1B5. In theory, it was possible that the glycosylated cargo were not anchored to the OM not through lipid A, but through a different type of lipid anchor, or even that it was attached to the cell surface without the need of a lipid anchor. In this study we showed for the first time that the T9SS cargo and A‐LS region is bonded to the *O*‐PS, and that the A*O*‐PS was in turn bonded to a core oligosaccharide containing Kdo. The defining feature of LPS in diverse Gram‐negative bacteria including *P. gingivalis* is the lipid A anchor along with its linkage to the core via one or more Kdo residues (Raetz and Whitfield 2002; Swietnicki and Caspi 2021). The identification of Kdo in our elucidated structures therefore strongly implies the presence of lipid A prior to the mild acid hydrolysis treatment of the sample.

In addition to the absence of phosphomannan, we were also unable to detect a core oligosaccharide consistent with the previously published A‐LPS structure (Paramonov et al. 2009; Paramonov et al. 2015). We are unable to explain the discrepancy since the methods used were very different. Besides the purification methods being substantially different, the NMR methods used by Paramonov et al are also very different to the methods used in the current paper. NMR requires high purity as it reports on the major product(s) of the sample, while LC‐MS methods work well on mixtures as the various compounds present can be separated both by the LC dimension and in the MS instrument. In addition, Paramonov et al used Western blotting to detect the reactivity of their phosphomannan sample to MAb‐1B5 (Paramonov et al. 2005). In our view, the positive reactivity to MAb‐1B5 indicated the presence of A‐LPS (as defined here). A‐LPS was likely a low‐level “contaminant” in their phosphomannan sample, as the purity of their sample was not well established despite multiple rounds of chromatography. Importantly, our method conclusively showed the bonding of T9SS cargo protein to A‐LPS molecules (as defined here) whereas no one has shown the bonding of phosphomannan to T9SS cargo proteins. In addition, supporting the original association of phosphomannan with MAb‐1B5 reactivity was the finding that dephosphorylation of their sample destroyed the MAb‐1B5 epitope (Paramonov et al. 2005). This can be explained by the conditions used for the “dephosphorylation” actually caused the deamidation of the SAGA sugar, an alteration which we have previously shown abolishes reactivity to MAb‐1B5 (Veith et al. 2020).

Regarding the limitations of this study, our analysis was not complete, with the possible presence of other compounds not being amenable to detection. Reasons for this could include inability to bind to the C18 column or being poorly ionized in the positive mode of the MS instrument. Additional structural studies are required to determine the full structure of the A‐LS including the specific sugars and linkages present, and also of the core oligosaccharide.

## 5 Conclusion

In this study we presented the first putative structure of A‐LPS that explains the substantial data associated with A‐LPS biosynthesis mutants. In so doing, we have suggested a correction to the original proposed structure of A‐LPS that was distinguished by its poly‐phosphomannan. Our findings conclusively establish that T9SS‐secreted cargo are covalently bonded to *O*‐PS via a single copy of A‐LS, and provide strong evidence that the connectivity continues to a novel core oligosaccharide containing Kdo which implies the presence of lipid A. Together, this connectivity provides a new definition of A‐LPS. This conclusion was shown to explain contradictions in the literature and provide a strong working model for A‐LPS biosynthesis. Our A‐LPS structure and biosynthesis model provides a clear pathway for future studies aimed at elucidating the structure in more detail, confirming the specific reactions of the glycosyltransferases, and exploring the implications of having two of the most important virulence factors of *P. gingivalis*, gingipains and LPS, covalently bonded together.

## Author Contributions

**Paul D. Veith:** conceptualization, investigation, funding acquisition, writing – original draft, methodology, validation, visualization, writing – review and editing, formal analysis. **Michael G. Leeming:** investigation, writing – review and editing, methodology. **Yu Yen Chen:** formal analysis, writing – review and editing. **Eric C. Reynolds:** funding acquisition, writing – review and editing, supervision.

## Ethics Statement

The authors have nothing to report.

## Conflicts of Interest

The authors declare no conflcits of interest.

## Supporting information

Supporting File 1

Supporting File 2
