# Supplementary material for: Cargo Secreted by the Type IX Secretion System of Porphyromonas gingivalis Are Tethered to O‐Lipopolysaccharides via a Pentasaccharide Linker
Source: Microbiologyopen. 2026 Apr 16;15(2):e70296. doi: 10.1002/mbo3.70296 (PMC13086638; doi:10.1002/mbo3.70296)
Supplement: Supplementary file 1 — Supporting File 1 [file MBO3-15-e70296-s002.pdf]

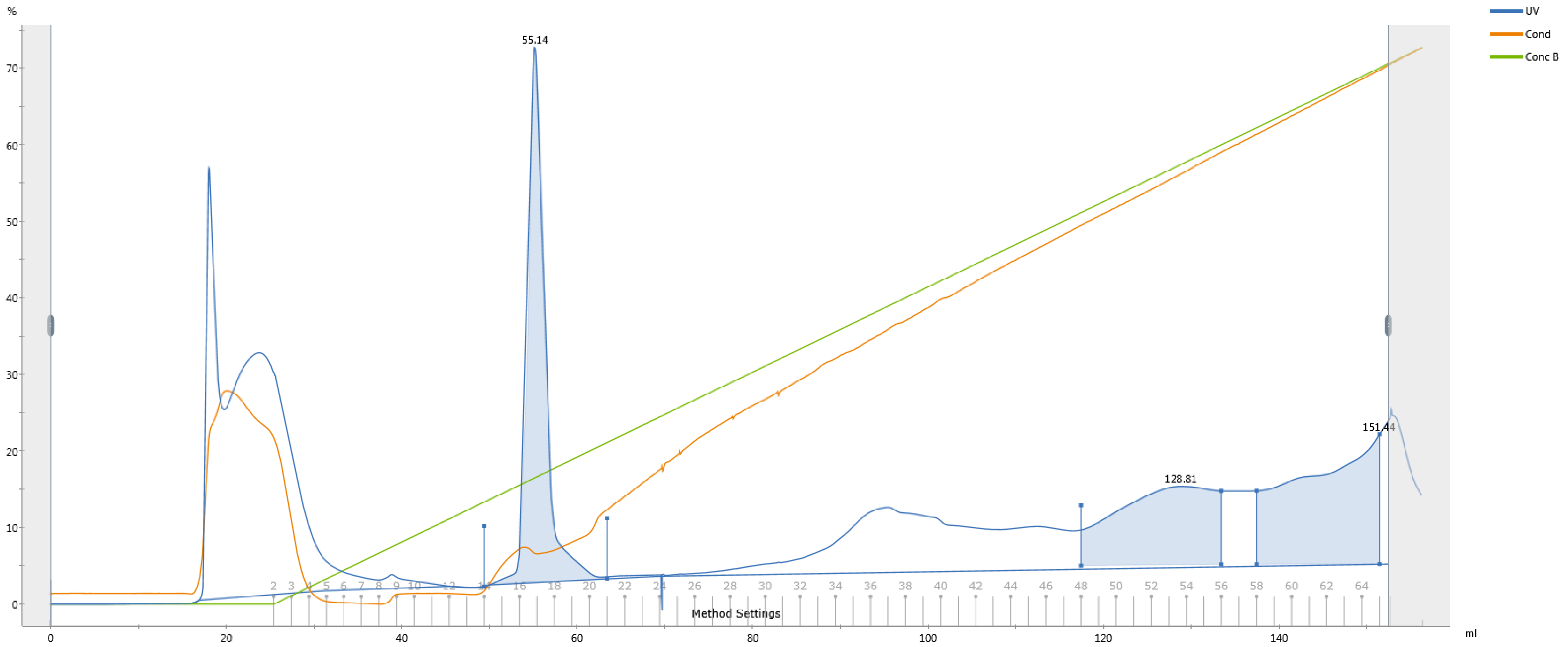

Figure S1: Anion exchange purification of LPS-modified cargo proteins. Samples enriched in outer membrane proteins were separated using a Q-Sepharose column in the presence of detergent. Fractions (2 mL) were collected as indicated, and selected ones were analysed by SDS-PAGE (Fig S2).

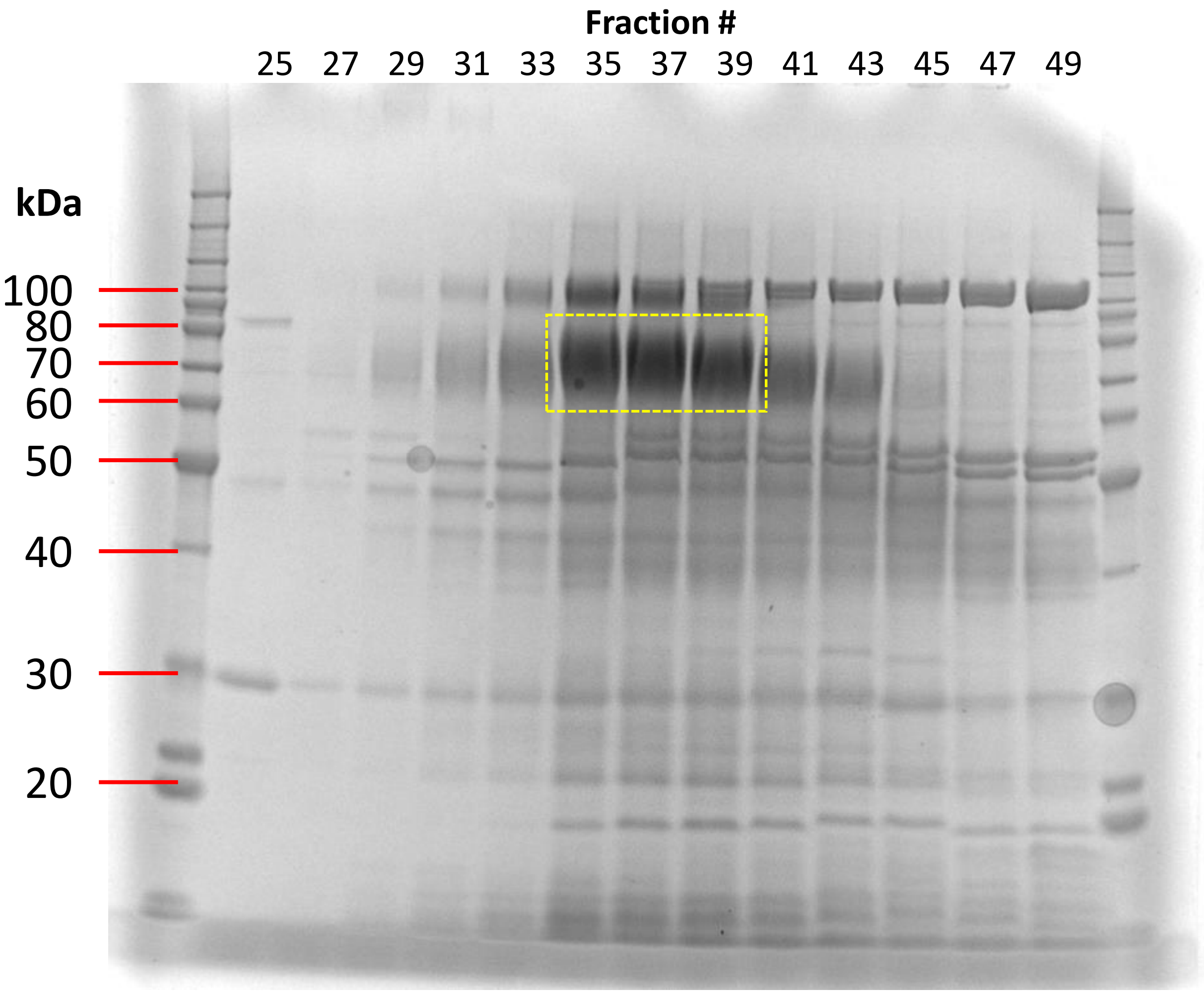

Figure S2: SDS-PAGE of Q-Sepharose fractions. The indicated fractions were separated on a 10% polyacrylamide 15-well gel and stained with Coomassie blue. Sample loading was according to the UV absorbance shown in Fig S1. 300 uL was used for fractions 25-31, while 100 uL was used for fractions 33-49. These volumes required concentration by TCA precipitation prior to redissolving in SDS sample buffer. The yellow box indicates the location of LPS-modified RgpB and TapA. Fractions 34-35 were pooled as “F1” and fractions 39-40 were pooled as “F2”.

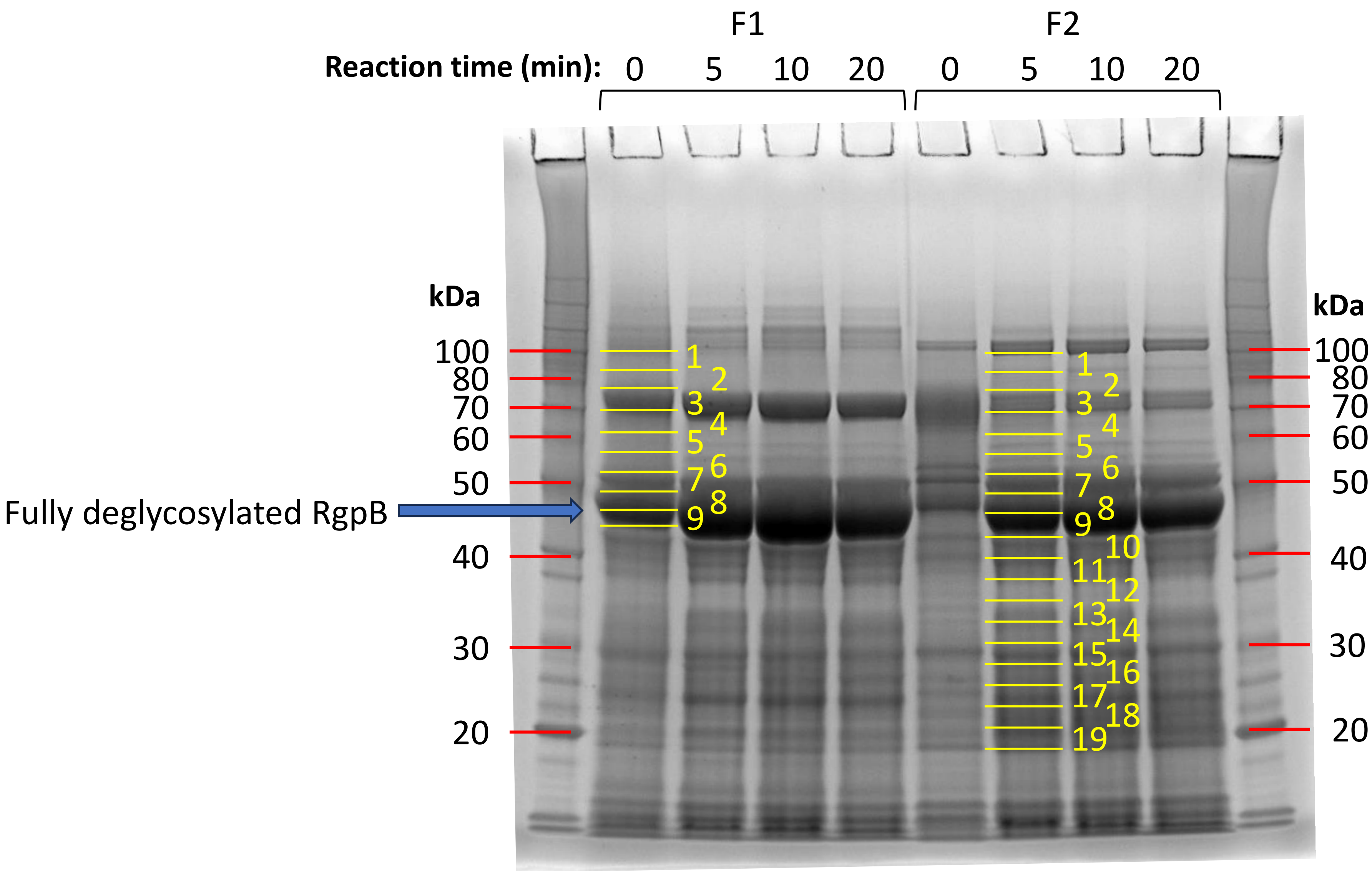

Figure S3: SDS-PAGE of deglycosylated F1 and F2 fractions. F1 and F2 were deglycosylated using TFMS with a reaction time ranging from 0-20 min as indicated and analysed by SDS-PAGE with Coomassie blue staining. Bands 1-9 were excised from the 0 min lane of F1 and digested with trypsin. Likewise, bands 1-19 were excised from the 5 min lane of F2 and digested with trypsin. The digested bands were then analysed by LC-MS/MS. Where the reaction time was 0 min, the TFMS was added to the sample, but the incubation step was omitted (see methods).

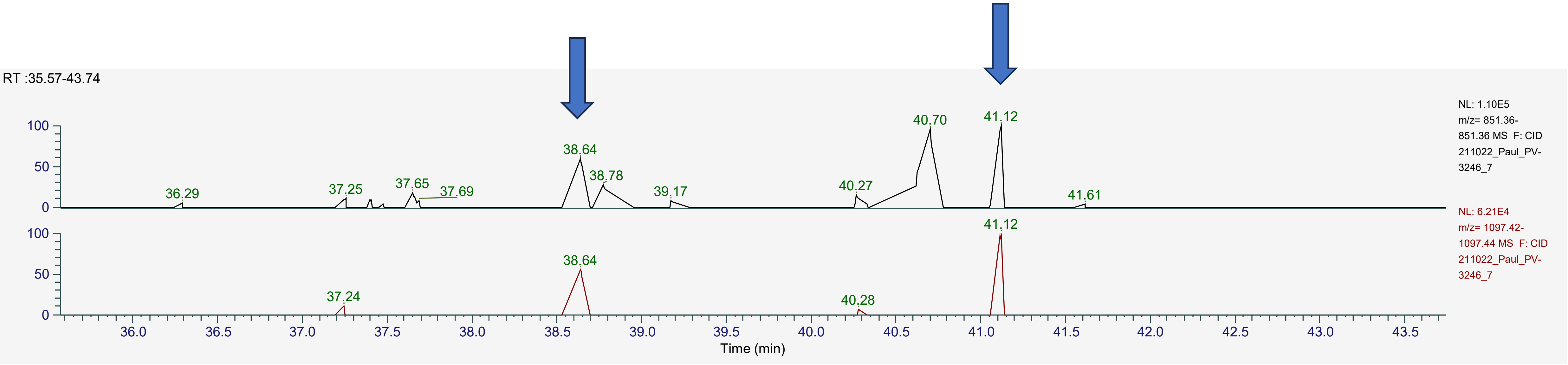

Figure S4: Extracted ion chromatograms (EICs). The EIC traces are from the LC-MS/MS run of Band 7 from F1 (Fig S3). A window from 36 to 43.5 min is shown. In the top trace, the abundance of CID ions at m/z 851.36 is shown, while in the bottom trace, the abundance of CID ions at m/z 1097.43 is shown. These ions correspond to the expected B_2 and B_3 ions of the glycosylated C-terminal peptide of RgpB (see main Figure 1). The peaks at 38.64 min and 41.12 min present in both traces indicate the likely presence of glycosylated C-terminal peptides of RgpB at those time points allowing the corresponding MS/MS spectra for these glycopeptides to be located.

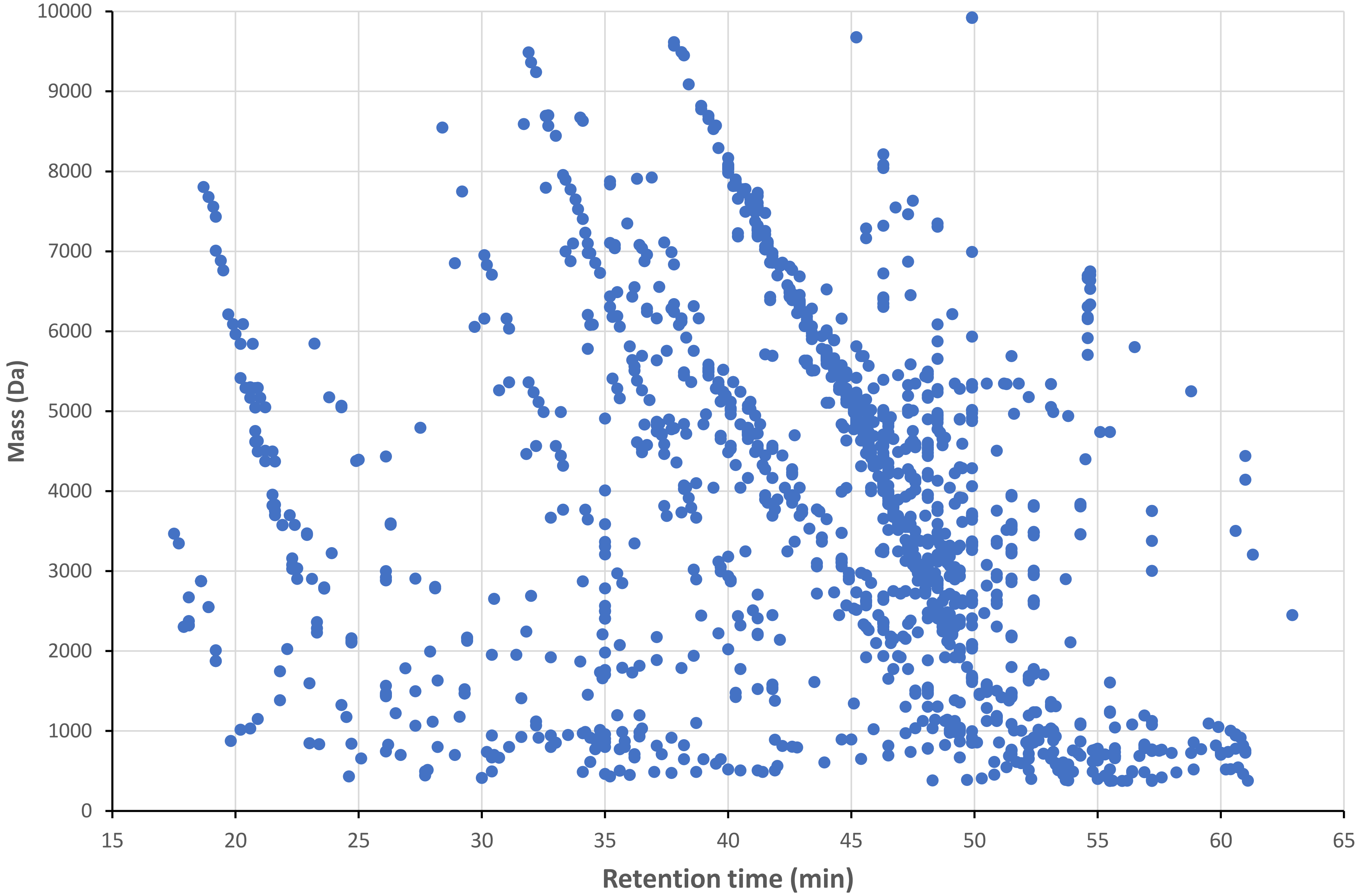

Figure S5: Mass versus retention time plot of detected compounds. The LC-MS/MS data for F2 was quantitated using Maxquant and processed in Excel. A mass-weighted intensity filter of 0.003 was applied to limit the number of compounds viewed. With this filter, 1568 compounds remained, and many could be seen arranged in diagonals which were found to represent the different series of polysaccharides.

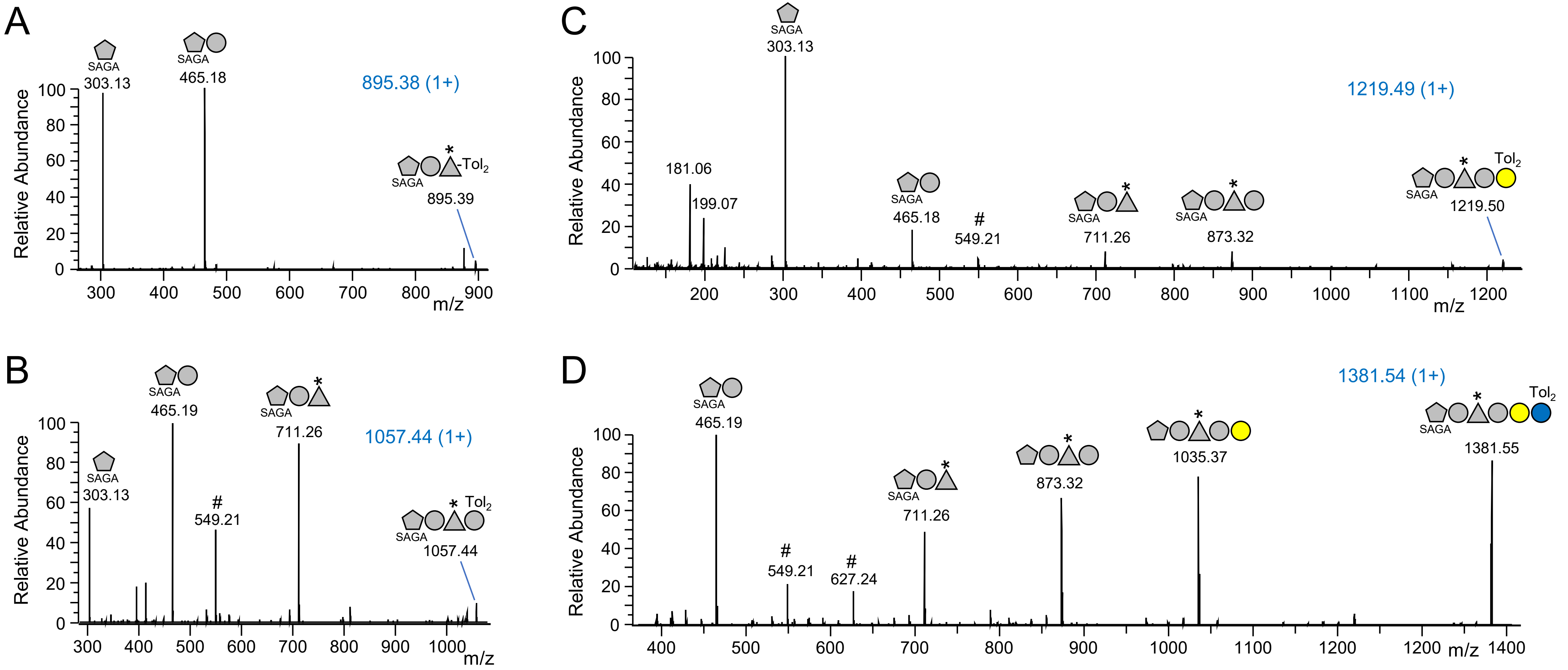

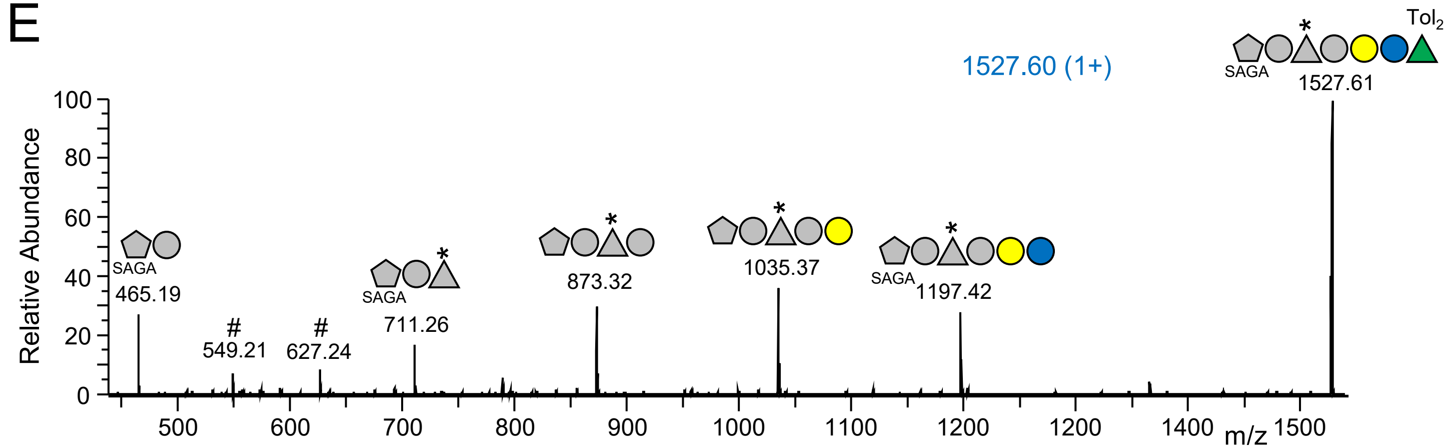

Figure S6: MS² spectra showing stepwise TFMS cleavage across the linkage region. The spectra are from partially deglycosylated and proteinase K digested T9SS cargo. For each spectrum (A-E), MS peaks are directly labelled with the assigned glycan. The m/z value of the precursor ion is shown at the top right of each spectrum in blue text. All fragment ions are singly-charged. A, B, D, E. CID spectra. C. Average of one HCD and one CID spectrum. #Indicates ions that may be produced by dHex scrambling.

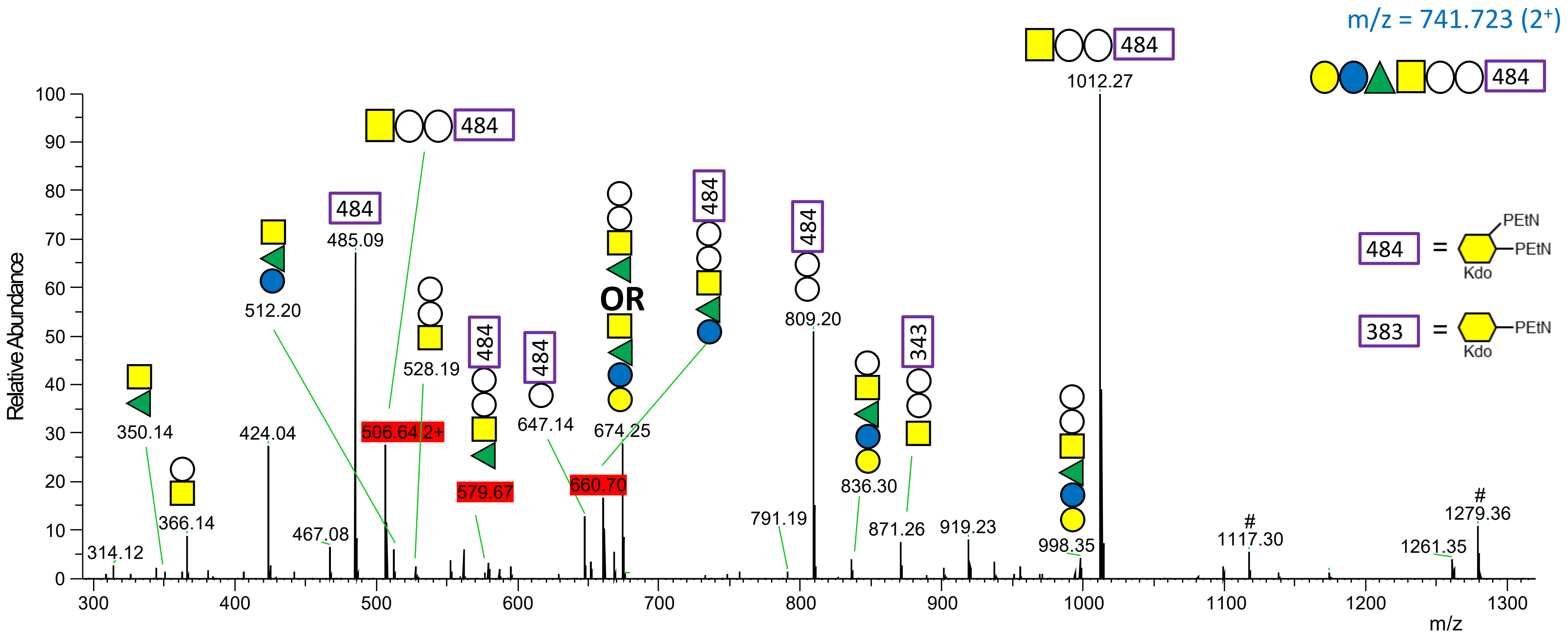

Figure S7: MS² spectrum of the core oligosaccharide (without hexosamine) linked to *O*-PS. The CID spectrum comes from the LC-MS/MS analysis of *a porT/wzzP* double mutant-derived sample after lipid A removal. The *m/z* value of the precursor ion is shown at the top right in blue text and the assigned glycan is shown underneath. All labelled fragment ions are single-charged except those shown highlighted in red which are double-charged. #Indicates ions that may be produced by dHex scrambling.

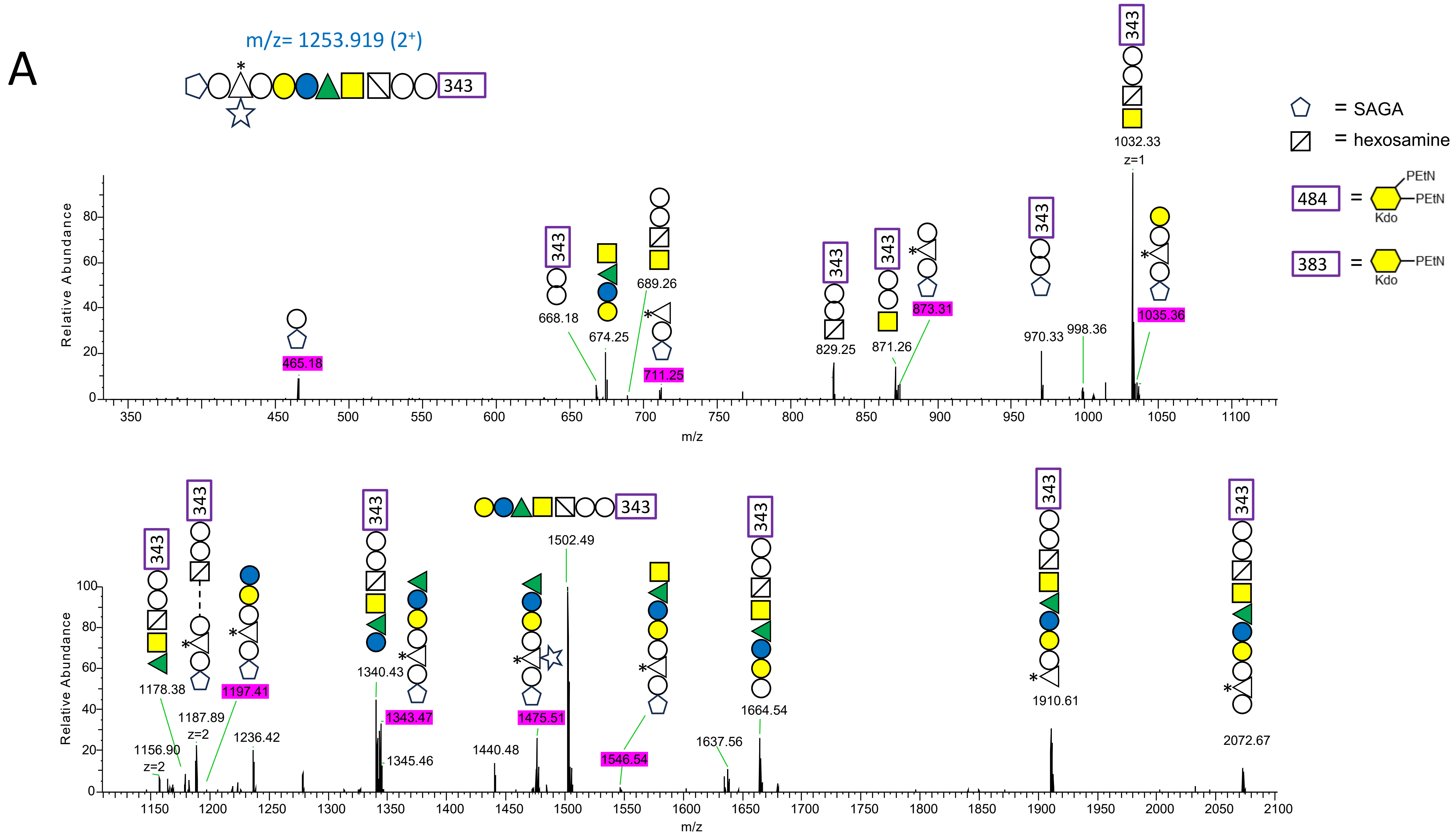
A
m/z= 1253.919 (2+)
343
= SAGA
= hexosamine
484
= PEtN PEtN Kdo
383
= PEtN Kdo
Relative Abundance
m/z
465.18
668.18
674.25
689.26
711.25
829.25
871.26
873.31
970.33
998.36
1032.33
z=1
1035.36
1156.90
z=2
1178.38
1187.89
z=2
1197.41
1236.42
1340.43
1343.47
1345.46
1440.48
1475.51
1502.49
1546.54
1637.56
1664.54
1910.61
2072.67

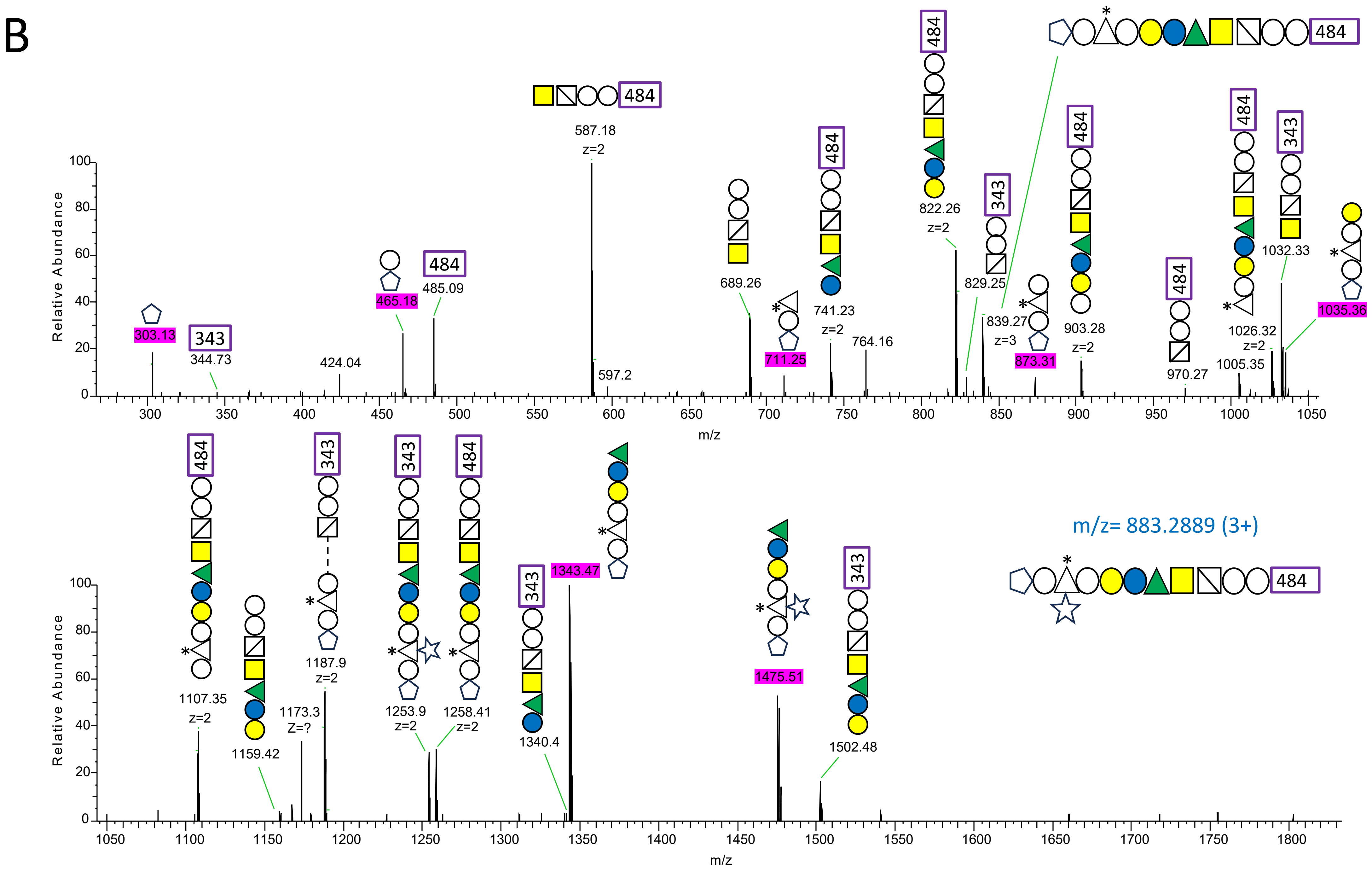
B
m/z= 883.2889 (3+)
Relative Abundance
m/z
484
343
303.13
344.73
424.04
465.18
485.09
587.18
z=2
597.2
689.26
711.25
741.23
z=2
764.16
822.26
z=2
829.25
839.27
z=3
873.31
903.28
z=2
970.27
1005.35
1026.32
z=2
1032.33
1035.36
1107.35
z=2
1159.42
1173.3
Z=?
1187.9
z=2
1253.9
z=2
1258.41
z=2
1340.4
1343.47
1475.51
1502.48

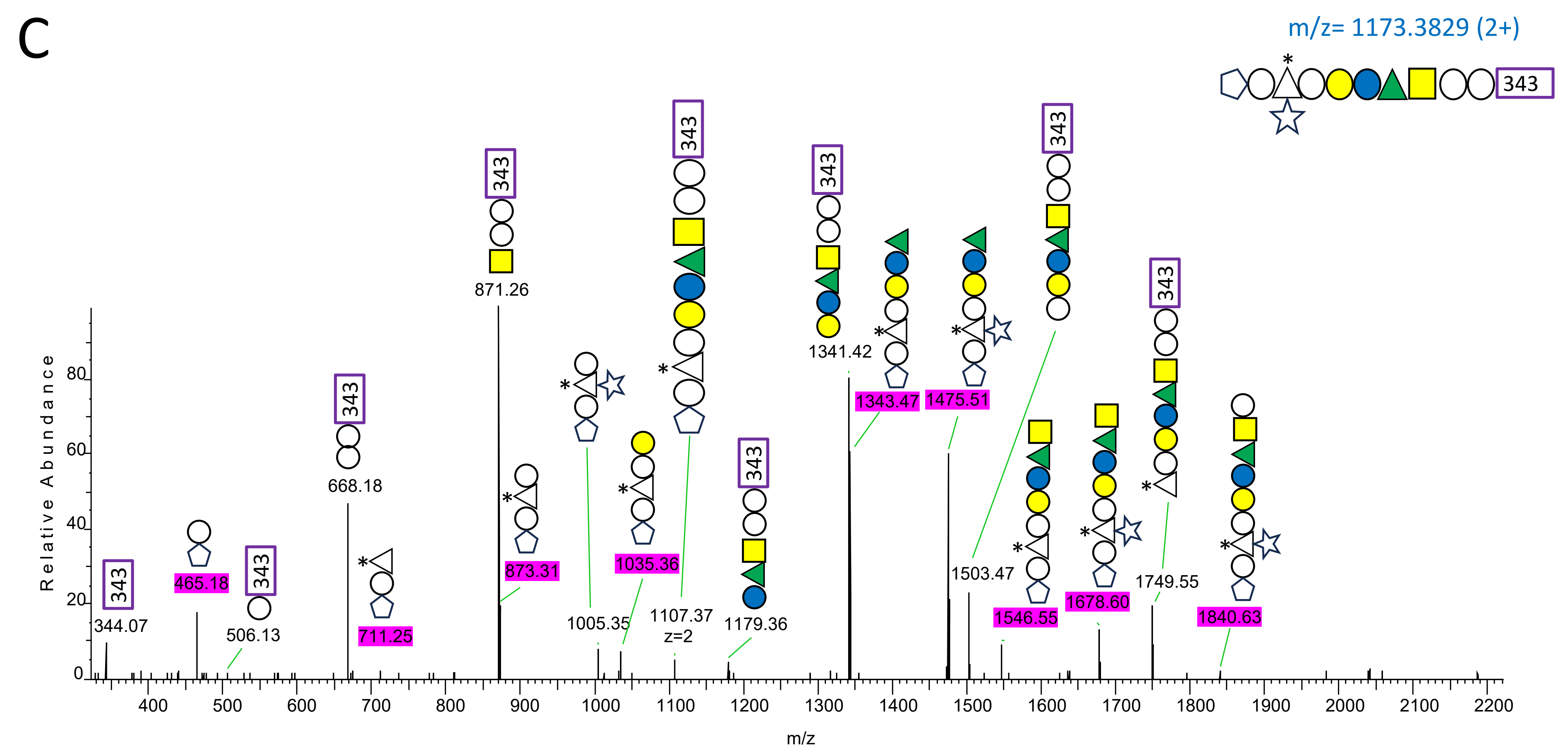

Figure S8: MS² spectra of the core oligosaccharide linked to *O*-PS and the A-linking saccharide. The CID spectra (A-C) come from the LC-MS/MS analysis of *a porT/wzzP* double mutant-derived sample after lipid A removal. The *m/z* value of each precursor ion is shown in blue text together with the structure of the assigned glycan. All labelled fragment ions are single-charged unless shown otherwise. Mass labels shown highlighted in pink correspond to the series beginning with SAGA (Y-ions).
